# Taming extreme morphological variability through coupling of molecular phylogeny and quantitative phenotype analysis as a new avenue for taxonomy

**DOI:** 10.1038/s41598-019-38875-2

**Published:** 2019-02-20

**Authors:** Tomislav Karanovic, Martin Bláha

**Affiliations:** 1 Hanyang University, Department of Life Science, Seoul, 133–791 South Korea; 2 University of South Bohemia, Faculty of Fishery and Protection of Waters, South Bohemian Research Centre of Aquaculture and Biodiversity of Hydrocenoses, Zátiší 728/II, 38925 Vodňany, Czech Republic

## Abstract

Identification of animals is often hindered by decoupling of phenotypic and molecular evolutionary rates. The *Acanthocyclops vernalis* (Fischer, 1853) complex is arguably the most problematic group of cyclopoids and possibly of all copepods, with diversity estimates based on morphology ranging from 2 to 34 taxa. We reconstructed their phylogeny based on one nuclear and three mitochondrial markers, revealing only four species in the Holarctic and always the following sister-species pairs: *vernalis–europensis* sp. nov. and *robustus*–*americanus*. Landmarks for quantitative shape analyses were collected from 147 specimens on five structures commonly used to delineate cyclopoids. Procrustes ANOVA showed small directional asymmetry in all datasets, but large sexual dimorphism in shape and size. Allometry was also highly significant. Principal component analyses of size-corrected data almost completely separated species in morphospace based on the last exopodal and endopodal segments of the fourth leg. These two structures showed the highest amount of covariation, while modularity could not be proven and a phylogenetic signal was only observed in one structure. Spinules and sensilla have a limited use in delineating species here. Calculating mean shapes and the extent of inter and intraspecific phenotypic variability opens new horizons for modern taxonomy.

## Introduction

Landmark-based geometric morphometrics (LBGM) is an emerging field of biology^1,2^, increasingly frequently employed in studies from the broader context of ecology and evolution^3^, biogeography^4^, and developmental biology^5^. Its high statistical power makes it suitable for detecting even subtle variation, such as asymmetries in morphological structures^1,6,7^. A quantification of variation invisible to the human eye makes LBGM a valuable tool in taxonomy, but so far it was used rarely and mostly for delineation of cryptic species^8–11^. However, it is still underexploited in this context^12^, and is yet to be used to solve taxonomic problems where too much variability is a major concern. In-depth analyses of morphological characters that failed in species delineation due to their variability are also lacking.

In copepod taxonomy few problems have received as much attention as the *vernalis*-complex, partly because of its wide distribution and wide range of ecological tolerances^13,14^ and partly because of its extreme morphological variability^15,16^. For example, the number and nature (spine or seta) of armature on the swimming legs, which in most copepods are diagnostic of different genera^17^, were often observed as asymmetries here^18,19^ (see also Supplementary Fig. S1). These cyclopoids are capable of surviving in a variety of freshwater habitats^20,21^, and they are extremely successful in disturbed habitats^13,22^. Their accessibility and eurytopicity made them model organisms for *in situ* and *in vitro* studies on general ecology^13,22–24^, disease vector control^25^, parasitology^26^, ecotoxicology^27^, development^28^, predatory behaviour^29^, swimming behavior^30^, karyology^31,32^, and chromatin diminution^33^. However, their taxonomy remains in a state of flux^18,34–36^, and since the first members were described 150 years ago^37–39^ more than 34 species and subspecies have been attributed at some stage to this complex^14,36^. Today, many of these names are considered synonyms by at least some researchers^14^, and at one point only two species were accepted as valid in Europe^20^. Negative *in-vitro* cross-breeding experiments between some North American populations suggested numerous possible species, although most of them are morphologically indistinguishable^16,40^. However, preliminary molecular studies in Europe and North America^41,42^ cast doubts on the cross-breeding results, but suggested other possible cryptic species.

Here we aim to resolve their taxonomy based on a multi-gene approach in combination with LBGM analyses of phenotypes. In addition to providing usable morphological characters for species delineation, we aim to investigate their range of variability, covariation, evolutionary signal, and modularity. Another important goal is to describe previously undescribed species using outputs from LBGM, and to provide a list of synonyms for known species. Most biological data lose value when not linked to a formal species name^43^, and taxa have to be named to benefit from any conservation program^44^. Unfortunately, most molecular research detecting cryptic species does not lead to species descriptions^45^. Due to typological analyses of phenotypes and a general lack of taxonomic expertise, descriptions of new species based solely on molecular characters are becoming more common^46,47^, which could pose new and unexpected challenges for taxonomy in general.

**Figure 1:**
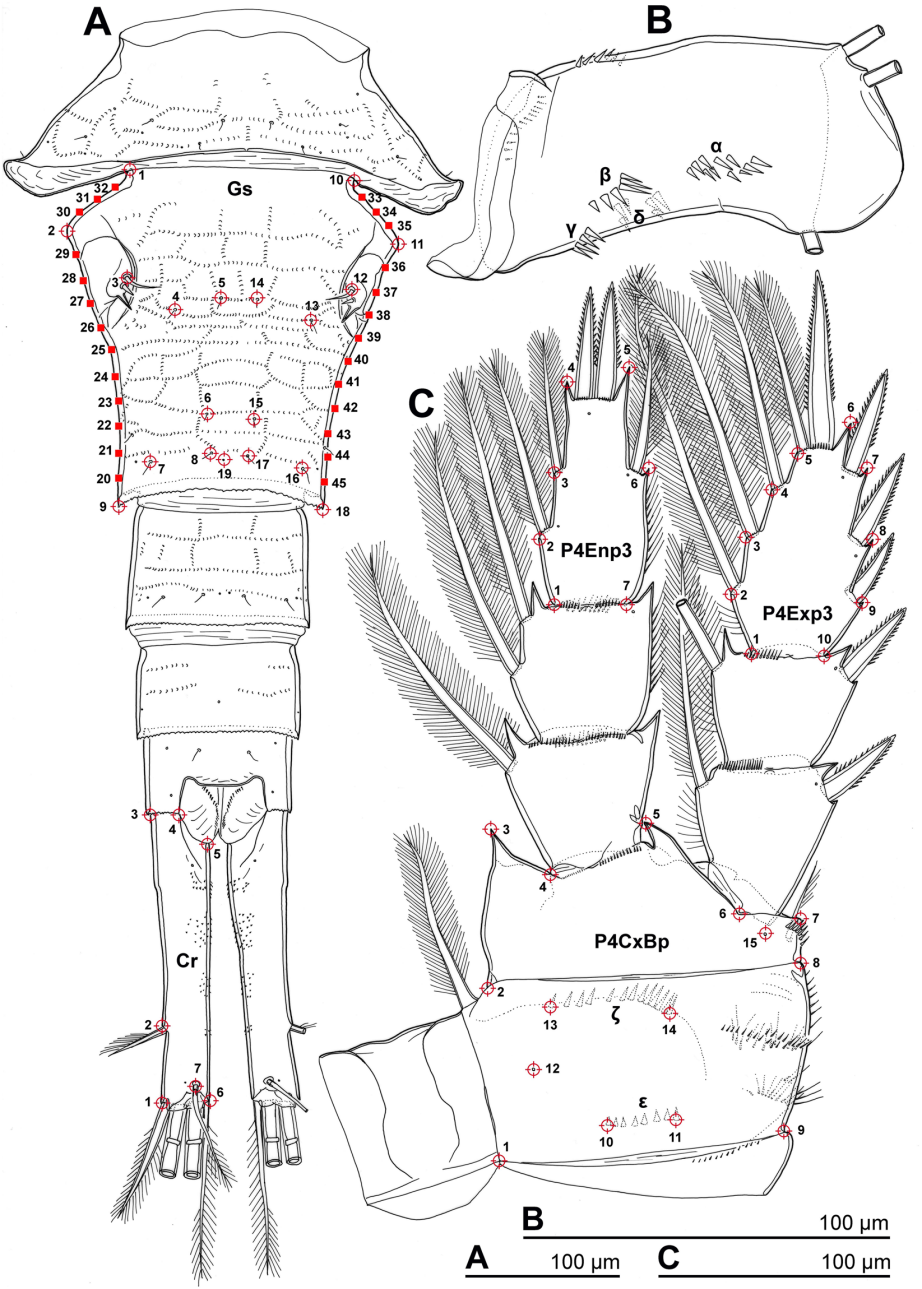
*Acanthocyclops europensis* sp. nov., morphological details of the holotype female. (**A**) Urosome, dorsal; (**B**) Basis of antenna, anterior; (**C**) Fourth swimming leg, anterior. Greek letters indicate rows of spinules counted. Red target symbols indicate positions of chosen landmarks and red squares indicate sliding landmarks for five rigid structures digitized for 2D geometric morphometric analyses: (Gs) Genital somite; (Cr) Caudal ramus; (P4Enp3) Third endopodal segment of the fourth leg; (P4Exp3) Third exopodal segment of the fourth leg; (P4CxBp) Coxa and basis (= protopod) of the fourth leg.

We hope to be able to understand why taxonomists struggled with the *vernalis*-complex and the characters they used, as lessons learned could potentially be applied broadly to small animals with difficult taxonomy. LBGM data were collected from five rigid structures (Fig. 1), all previously considered important for species delineation in this complex and other cyclopoids: female genital somite (Gs), female and male caudal rami (Cr), protopod (coxa and basis) of the fourth leg (P4CxBp), and third exopodal (P4Exp3) and endopodal (P4Enp3) segments of the same leg.

We also wanted to test the suitability of micro-characters, such as cuticular ornamentation (spinules and pits) and cuticular organs (pores and sensilla) for species delineation. Spinules on the antennal basis and the fourth leg coxa have been used widely in cyclopoid species descriptions^48^, but so far without any robust tests of intraspecific variability. This is important because several recently described species from the *vernalis*-complex have been based mostly or exclusively on these characters^36^. The logic behind using spinules on the fourth leg is based on the assumption that they might serve as species-recognition structures^48^. Distribution of pores and sensilla on somites, on the other hand, have recently been studied and used successfully to delineate species in several copepod groups^11,12,49^.

## Results

### Molecular phylogeny

**Figure 2:**
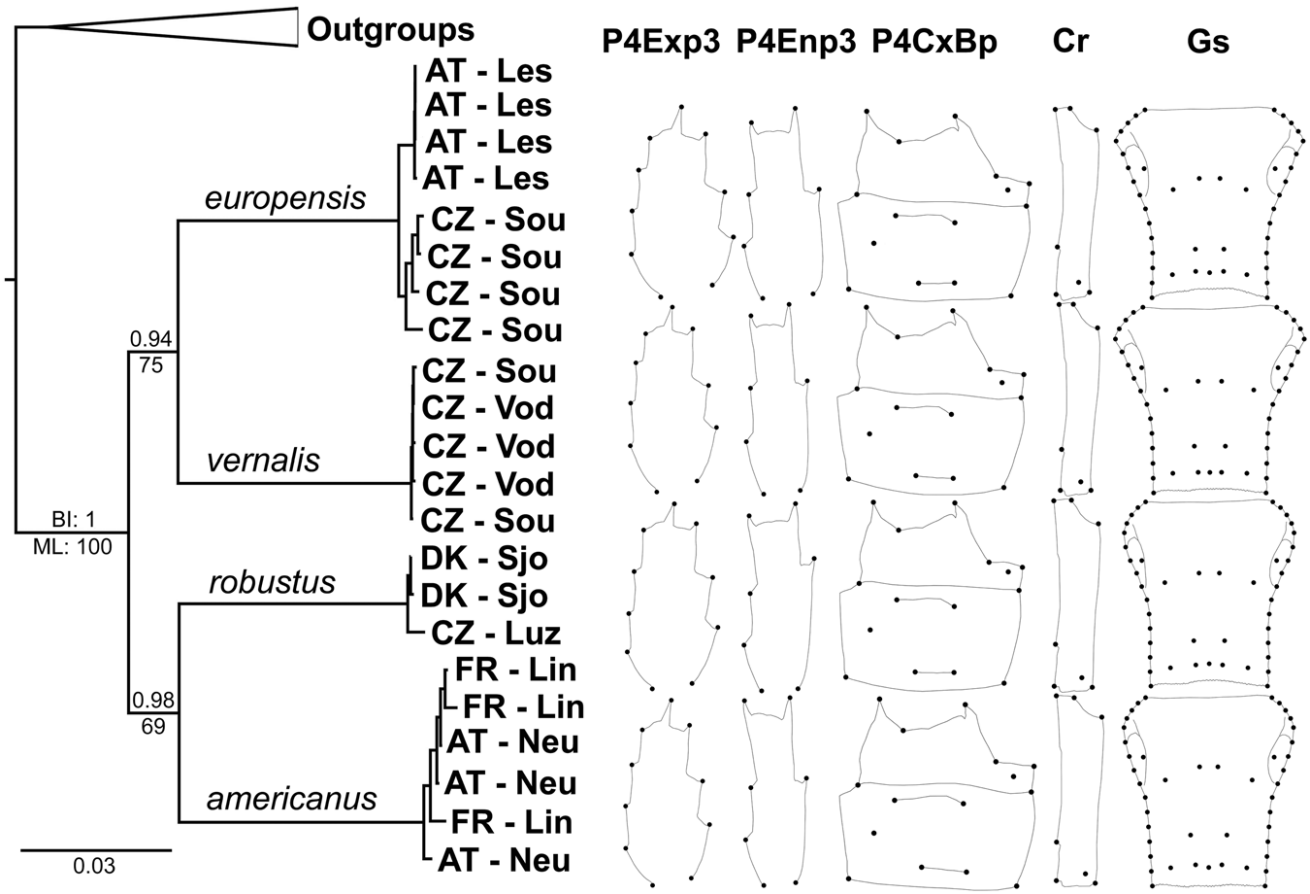
Bayesian inference (BI) tree resulting from the concatenated dataset (CytB + 12S + ITS-1), depicting relationships of four *Acanthocyclops* species, and warped outlines of five female morphological structures. The outlines have been scaled to the same centroid size and represent size-uncorrected mean shapes based on digitised landmarks (Fig. 1). Maximum likelihood (ML) bootstrap values and BI posterior probabilities are displayed below and above branches respectively. Specimen codes are a combination of a two-letter ISO country code and a three-letter locality code (Supplementary Table S6).

The concatenated dataset (CytB + 12S + ITS-1) consisted of 1,003 base pairs after GBlock adjustment, containing 240 variable sites of which 237 were parsimony informative. Reconstructed phylogeny supported the monophyly of *Acanthocyclops* Kiefer, 1927 species with the maximum bootstrap value and posterior probability (Fig. 2). Tree topology did not differ between the two methods employed, suggesting four deeply divergent clades and the same sister-species pairs. Bootstrap support for these pairs was moderate (75% and 69%), while posterior probabilities were high (0.98 and 0.94). Specimens from the same population clustered together in *europensis* and *robustus*, but not in the other two species. Tree topology based on COI was the same (Supplementary Fig. S2), the ingroup was again supported with the highest bootstrap value and posterior probability, but the support for the *europensis-vernalis* clade was slightly lower (bootstrap value 73%; posterior probability 0.92), and the support for the *robustus-americanus* clade was slightly higher (bootstrap value 69%; posterior probability 1).

Average interspecific distances were very high for all four genes (Supplementary Table S1). Excluding outgroups, they were 22%–38% for CytB; 20%–26% for 12S; 9%–15% for ITS-1; and 35%–66% for COI. Average intraspecific distances were low, with maximum values not exceeding 1% for either CytB, 12S, or ITS-1. Those for COI were slightly higher: *robustus* 0%; *americanus* 1%; *europensis* 2%; and *vernalis* 7%.

### Patterns of morphological variation

All 84 landmarks (LMs) were unquestionably homologized based on their nature (pore, sensillum, spinule, seta, cuticular corner) and/or relative position (Fig. 1). Not all specimens were examined for all structures because of abnormalities or asymmetries (Supplementary Fig. S1), the most common being only two outer spines on P4Exp3. Out of 22 *europensis* females from Lesach (Austria) only seven had three outer spines on both legs; out of 15 *americanus* females preserved in alcohol five had two spines on both legs and two were asymmetric; out of 33 *americanus* males preserved in alcohol only one was asymmetric and eight had two outer spines on both legs. In other populations only several specimens were observed with abnormal P4Exp3; abnormalities on P4Enp3 and P4CxBp were rare (Supplementary Fig. S1).

Procrustes ANOVA analyses for P4Enp3 showed that digitizing error represented less than 10% of imaging error, which in turn contributed less than 10% to fluctuating asymmetry of shape (Table 1). Digitizing error was thus considered negligible and was not evaluated for other structures. For all other structures imaging error contributed less than 10% to fluctuating asymmetry of shape and considerably less in size. Directional asymmetry was small and statistically insignificant (or barely significant) for size in almost all datasets, while it was significant for shape in all datasets, except for GsCo. It was largest for Cr (F = 9.31) and Gs (F = 5.07). Sexual size dimorphism was large and highly significant (P < 0.0001) for all four structures scored for both sexes. Sexual shape dimorphism was smaller, but still highly significant in all cases, being from 13 (P4Exp3) to 56 (Cr) times larger than individual variability. Therefore, we calculated mean shapes for each structure separately for females (Fig. 2) and males (Supplementary Fig. S2). Compared to sexual dimorphism, the effect of locality contributed little to overall variability, and more so in size than shape, but it was significant in all datasets (P ≤ 0.0005), except for GsCo. The effect of species was the largest contributor to shape variation, with the Goodall’s F critical value ranging from just over 14 (Gs) to more than 200 (P4Enp3). It was also an important contributor to size variation, and highly significant (P < 0.0001) in all cases both for shape and size.

**Table 1:** Shape and size variation inferred by a one–way nonparametric ANOVA with randomized permutation procedure (10,000 iterations).

		P4Exp3		P4Enp3		P4CxBp		Cr		Gs		GsCo		P4Bp	
		F	P	F	P	F	P	F	P	F	P	F	P	F	P
ANOVA Shape	Species	104.14	<0.0001	204.99	<0.0001	29.66	<0.0001	49.54	<0.0001	14.20	<0.0001	24.79	<0.0001	55.04	<0.0001
	Locality	1.93	<0.0001	1.82	0.0005	2.00	<0.0001	4.67	<0.0001	1.99	<0.0001	0.78	0.8517	2.33	<0.0001
	Sex	13.61	<0.0001	18.77	<0.0001	16.78	<0.0001	56.79	<0.0001	n/a	n/a	n/a	n/a	7.67	<0.0001
	Individual	3.90	<0.0001	4.42	<0.0001	2.34	<0.0001	4.78	<0.0001	4.04	<0.0001	2.18	<0.0001	2.86	<0.0001
	Directional asymmetry	1.98	0.0113	4.77	<0.0001	2.53	<0.0001	9.31	<0.0001	5.07	<0.0001	1.74	0.0752	2.83	0.0008
	Fluctuating asymmetry	11.14	<0.0001	13.98	<0.0001	19.81	<0.0001	15.35	<0.0001	10.49	<0.0001	46.42	<0.0001	10.73	<0.0001
	Imaging	n/a	n/a	10.51	<0.0001	n/a	n/a	n/a	n/a	n/a	n/a	n/a	n/a	n/a	n/a
ANOVA Size	Species	111.30	<0.0001	54.43	<0.0001	38.1	<0.0001	29.15	<0.0001	22.93	<0.0001	32.22	<0.0001	45.53	<0.0001
	Locality	8.82	<0.0001	10.34	<0.0001	9.05	<0.0001	10.43	<0.0001	17.76	<0.0001	13.96	<0.0001	8.29	<0.0001
	Sex	214.28	<0.0001	244.05	<0.0001	513.25	<0.0001	351.33	<0.0001	n/a	n/a	n/a	n/a	485.19	<0.0001
	Individual	88.84	<0.0001	72.18	<0.0001	200.59	<0.0001	86.97	<0.0001	732.95	<0.0001	511.91	<0.0001	122.43	<0.0001
	Directional asymmetry	0.12	0.7328	0.10	0.7554	4.01	0.0471	0.49	0.4830	n/a	n/a	n/a	n/a	21.00	<0.0001
	Fluctuating asymmetry	31.86	<0.0001	36.02	<0.0001	22.71	<0.0001	54.17	<0.0001	n/a	n/a	n/a	n/a	18.15	<0.0001
	Imaging	n/a	n/a	10.92	<0.0001	n/a	n/a	n/a	n/a	n/a	n/a	n/a	n/a	n/a	n/a

Abbreviations for morphological structures (datasets) as in Fig. 1. In addition to five full datasets, two datasets with a reduced number of landmarks were also examined (GsCo and P4Bp). Only Goodall’s F critical values (F) and probabilities of finding a random value larger than the observed value (P) are shown. Only the symmetric component is shown for the datasets with object symmetry (Gs and GsCo), which do not include males, thus no effect for sex (n/a). Digitising error was only measured for P4Enp3.

Warped outlines (scaled to the same centroid size) of female (Fig. 2) and male (Supplementary Fig. S2) structures were obtained by plotting raw Procrustes coordinates onto phylogeny. They represent mean value (mean of the symmetric component for Gs) of shape variation with allometry, based on landmarks in this study. When used for species delimitation it should be noted that shape changes are less reliable the further they are from landmarks, and that there are large interspecific size differences. For allometry analysis see Supplementary Note.

### Diversification in morphospace

**Figure 3:**
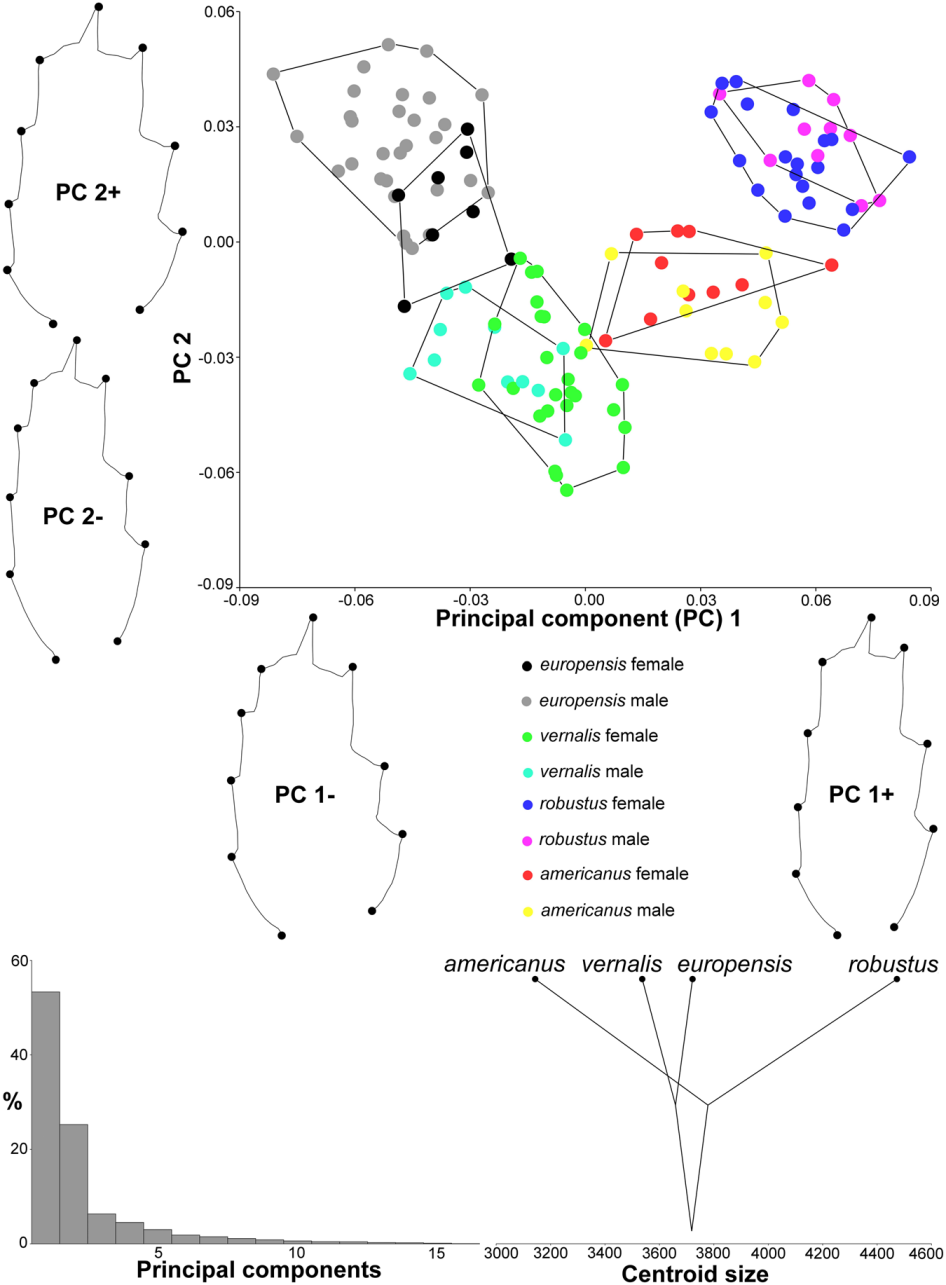
Graphical visualisation of the principal component analysis based on ten landmarks in the size-corrected P4Exp3 dataset (Fig. 1) for 136 specimens from seven localities (Supplementary Table S7). Scatter plot shows delimitation of species and sexes as convex hulls in morphospace defined by the first two eigenvectors (PCs). Warped outlines show shape changes at the observed extremes. Graph shows percentages of the total variance for each PC. Cladogram is the projection of phylogeny (Fig. 2) onto centroid size for each species mean value.

Principal component analysis (PCA) of P4Exp3 (Fig. 3), based on the covariance matrix of regression residuals (size-corrected symmetric component of shape, averaged by individual), revealed that nearly 80% of variability could be explained by the first two eigenvectors (Supplementary Table S2) and that in morphospace, explained by these eigenvectors, there is almost no overlap between species. The first eigenvector separates *europensis* and *vernalis* in the negative part from *robustus* and *americanus* in the positive part. This can be visualised by warped outlines as shape changes from wide segments in the negative part to narrow segments in the positive part. The second eigenvector mostly separates *vernalis* and *americanus* from *europensis* and *robustus*, with shape changes similar to those for the first eigenvector but reversed in sign. Hull surfaces are comparable for all species, despite differences in sample sizes, and sexual dimorphism in shape is more pronounced in *europensis* and *vernalis* than in *robustus* and *americanus*. Centroid size mapped onto phylogeny shows that this structure is largest in *robustus* and smallest in *americanus*.

**Figure 4:**
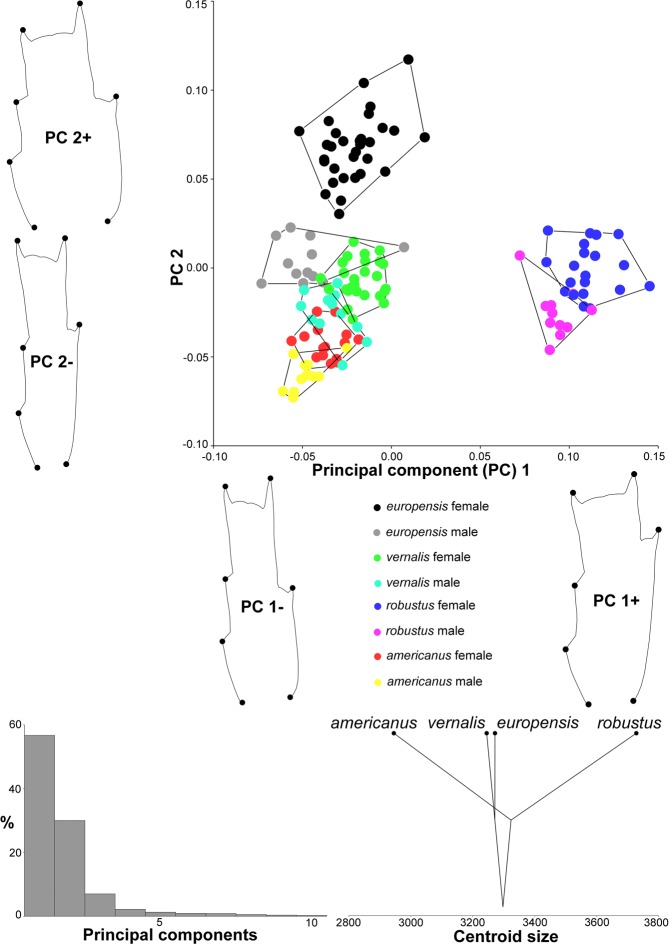
Graphical visualisation of the principal component analysis based on seven landmarks in the size-corrected P4Enp3 dataset for 146 specimens from seven localities. Details as in Fig. 3.

PCA analysis of P4Enp3 (Fig. 4) shows that nearly 90% of variability could be explained by the first two eigenvectors (Supplementary Table S2). There is very little overlap between species; no overlap between females and little overlap between males of *vernalis* and *americanus*. The shape changes are more pronounced than in P4Exp3 (0.15 vs. 0.09). The first eigenvector separates *robustus* from the other three species, with shape changes from distally inserted outer armature element (LM6; Fig. 1C) to this element inserted in line or slightly proximal to distal-inner element (LM3). The second eigenvector explains shape changes from narrow to wide segments, with relative shortening of the distal section. Intraspecific variability and sexual dimorphism are largest in *europensis*, but sexual dimorphism is more pronounced in P4Enp3 than in P4Exp3 in all species.

In other datasets the first two eigenvectors describe less variability, ranging from about 46% (P4CxBp) to 65% (Cr) (Supplementary Table S2). The separation of species in morphospace is also less pronounced. P4CxBp (Supplementary Fig. S3) fails to separate females of *europensis* and *vernalis*, but not males. Cr (Supplementary Fig. S4) fails to separate species (although their centres are clearly different) mostly because the first eigenvector (nearly 40% of variability) separates females from males; this is the only dataset where centroid size does not suggest *robustus* as the largest species, although *americanus* is still the smallest. Gs (Supplementary Fig. S5) also fails to separate species, but reveals that larger species (*robustus* and *europensis*) are also more variable than smaller species (*americanus* and *vernalis*) even after size correction; most shape changes are associated with the length of this somite and shape of the anterior expansion (rounded vs. angular). Analysis of only sensilla and pores on Gs (GsCo; Supplementary Fig. S6) produces hulls of more comparable size, separates species better, and completely separates sister-species: *europensis* and *vernalis* by the first eigenvector, *robustus* and *americanus* by the second.

### Covariation

**Figure 5:**
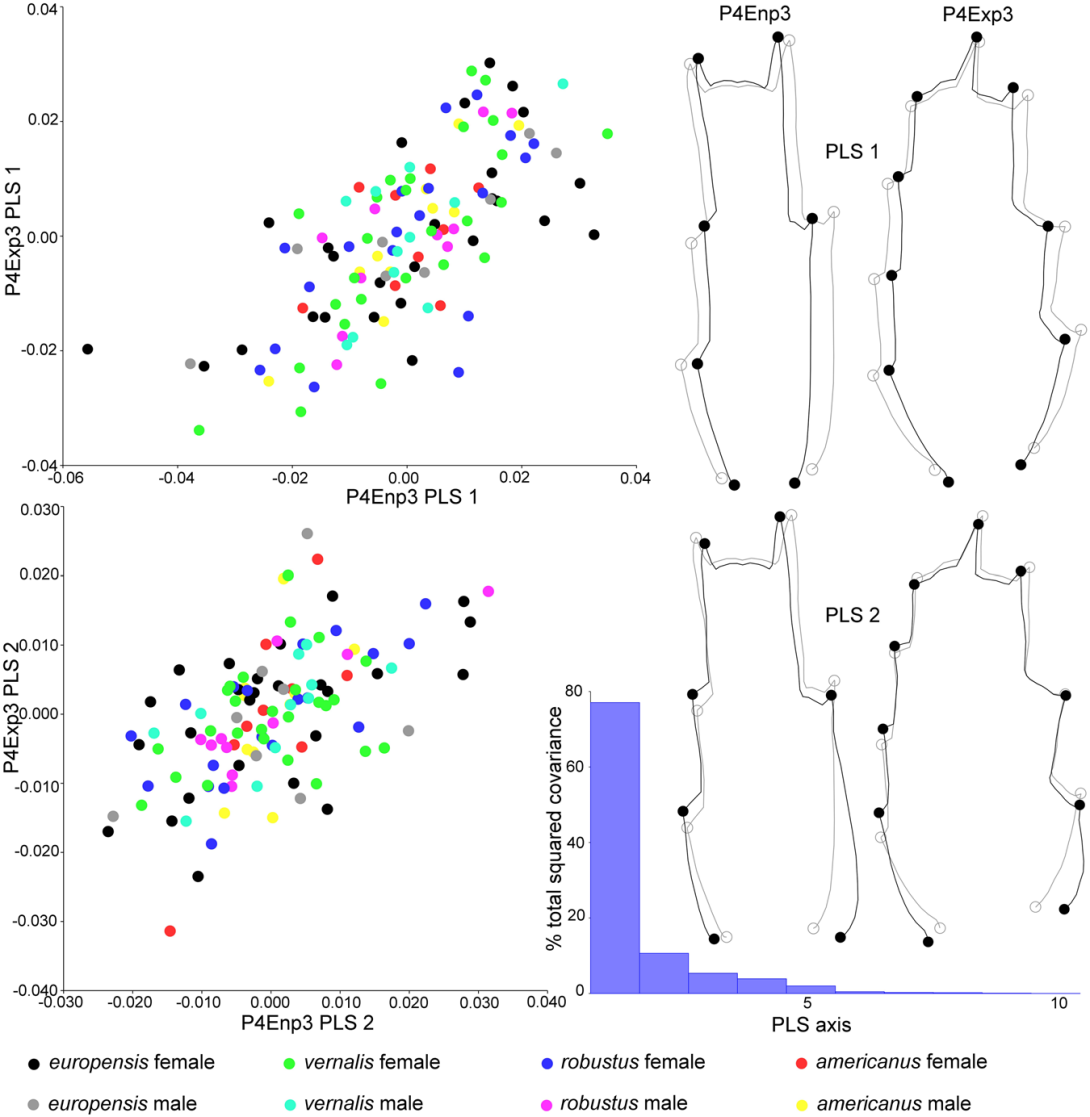
Graphical visualisation of covariation between P4Exp3 and P4Enp3, estimated from the partial least squares (PLS) analysis, pooled by species and sex. Scatter plots (left) show first two PLS axes, and warped outlines (right) show corresponding changes (black) from the mean shape (grey). Graph shows percentages of total covariance for each PLS axis. For RV coefficients and P-values see Supplementary Table S3.

The overall strength of association between datasets, as estimated by RV coefficient from partial least squares (PLS) analysis, was highest between P4Exp3 and P4Enp3 (RV = 0.3035; P < 0.001) (Supplementary Table S3). It was also highly structured, with the first PLS axis explaining nearly 80% of covariation. The shape changes for this axis (Fig. 5) indicate that when P4Enp3 becomes narrow so does P4Exp3; however, the scatter plot shows considerable noise. The noise is even higher for the second PLS axis (10% of covariation), and shape changes involve widening of the base for both structures.

RV coefficients were smaller for other pairs, and were not significant for P4Exp3-Gs and P4CxBp-Cr, while the covariation between P4Exp3 and Cr was barely significant (P = 0.0450). Of those that were significant, the strongest covariation was recorded for Gs-Cr (RV = 0.1201); RV values for P4Exp3-P4CxBp (0.115) and P4Enp3-P4CxBp (0.0981) were low.

Data integration within structures was higher, both when estimated from RV coefficients for different partitions of landmarks within configurations (not shown) and from the percentage of variation in the first few eigenvectors from PCA (Supplementary Table S2). In all datasets, except for P4CxBp, 90% of variability was explained by the first seven eigenvectors, and in P4Enp3 the same was explained by the first three. In all cases, except in Cr, size-corrections resulted in better integrations. Integration within structures was also high when estimated from matrix correlation between individual variation and fluctuating asymmetry (Supplementary Table S4), and it was significant in all datasets when pooled by species and sex (P ≤ 0.039), except for the symmetric component of GsCo without diagonal blocks (P = 0.064). Matrix correlation was significant even when not pooled in all datasets (P ≤ 0.0027), except for Cr and GsCo.

### Modularity

**Figure 6:**
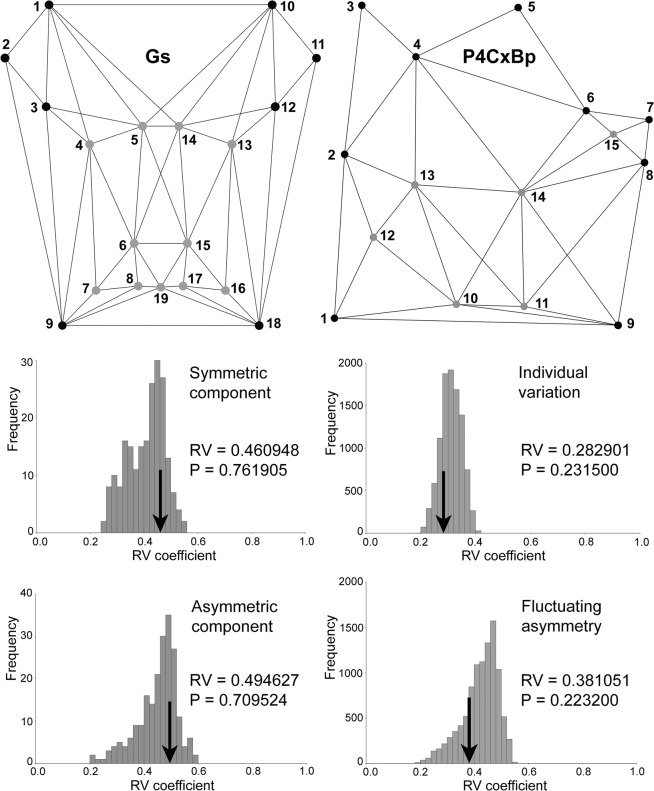
Modularity hypotheses tested for a selection of landmarks in the Gs dataset (left) and all landmarks in the P4CxBp dataset (right) (see Fig. 1). In both adjacency graphs one module includes landmarks representing ornamentation (cuticular organs in Gs and cuticular organs and spinules in P4CxBp; grey dots), while the other module includes landmarks representing cardinal points (black dots). Graphs show the RV coefficients (arrows) for the distribution of 10,000 alternative contiguous and non-contiguous partitions of landmarks (histograms).

Modularity hypotheses were tested for Gs on a selection of 19 LMs (excluding sliding ones; see methods). RV coefficients were high and not significant for a partition of landmarks that grouped all cuticular organs in one subset, both for the symmetric and asymmetric components (Fig. 6). This was unexpected because cuticular organs produced different delimitation of species in PCA than when used in combination with other LMs (Supplementary Figs S5,S6). Partition of LMs into anterior and posterior subsets, corresponding to second and third urosomites, also failed to produce significant RV coefficients (Supplementary Fig. S7), which suggests a strong integration in this complex structure.

Modularity was tested for P4CxBp with partitions that included all ornamentation (two pores and four LMs based on spinules) in one sub-set, but the RV coefficients were again relatively high for the distribution of 10,000 alternative contiguous and non-contiguous partitions of LMs and not significant, both for individual variation and fluctuating asymmetry (Fig. 6). The results did not change much for any modularity hypotheses when only contiguous partitions were considered (not shown). For evolutionary integration analysis see Supplementary Note and Supplementary Fig. S8.

### Spinules

To test usability of spinules for species delimitation, we counted them in four rows on the antenna (Greek letters in Fig. 1B), two rows on the fourth leg (Fig. 1C), and along the ventral margin of the labrum. While mean values differed between species in all antennal rows in both sexes, large variability limits their use in taxonomy, although some could be used for certain species pairs (Supplementary Fig. S9): row β distinguishes both sexes of *americanus* from *robustus*, and row δ is almost as useful in distinguishing *vernalis* from *robustus*. The fourth leg was even less useful, with females showing almost no differences in mean values in row ε, and larger species (*europensis* and *robustus*) showing more spinules in row ζ; interspecific differences in labral teeth were equally inadequate (Supplementary Fig. S10).

**Figure 7:**
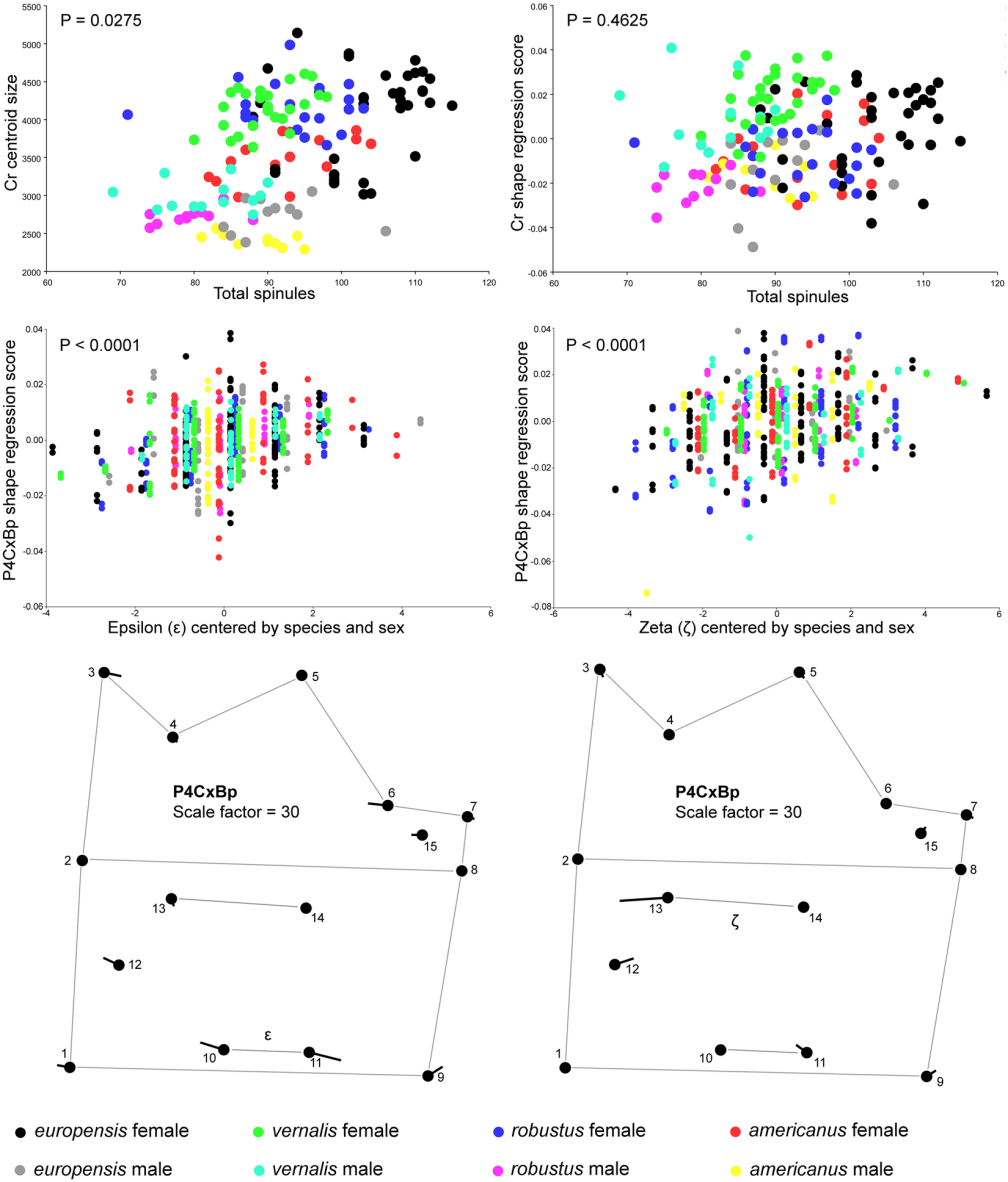
Covariation between the number of spinules and size and shape. Regression of the total number of spinules (Fig. 1, Supplementary Figs S9,S10) onto Cr centroid size (top left scatter plot) and Cr shape (top right scatter plot). Regression of the number of spinules in the proximal row (ε) onto P4CxBp shape, pooled by species and sex (bottom left scatter plot), and the shape change associated with it (left wireframe diagram, scaled 30 times). Regression of the number of spinules in the distal row (ζ) onto P4CxBp shape, pooled by species and sex (bottom right scatter plot), and the shape change associated with it (right wireframe diagram, scaled 30 times).

To test the hypothesis that the number of spinules simply increases with body size we regressed total spinules for each specimen onto centroid size and shape of Cr: the correlation was significant for size but not for shape (Fig. 7). The results did not change when centroid sizes of other structures were used (not shown). However, regression of the number of fourth leg spinules onto centroid size and shape of P4CxBp showed significant correlation for shape (Fig. 7) but not for size (not shown). Most shape changes occur in LMs associated with each row, but interestingly the change is equally spread among both landmarks in row ε, while it is mostly concentrated in the inner landmark for row ζ. These shape changes, however, are minute (scaled 30 times in Fig. 7) and would be useless as taxonomic characters.

### Taxonomy

*Acanthocyclops europensis* sp. nov. (Fig. 1, Supplementary Figs S11–S17). Holotype: female dissected on two slides (WAM C55924) from forest pool in Sousedovice, Czech Republic (type locality; Supplementary Table S6). Paratypes: 27 females and 12 males dissected on one slide each (C55925-63) from type locality; nine females dissected on one slide each (C55964-72) from Lesach, Austria; seven females and five males on one SEM stub (C55973) from type locality; 11 females and five males in two vials (C55974-5) from type locality; and 13 females in one vial (C55976) from Lesach. Etymology: the species name is an adjective for place (Europe), made with the Latin suffix “-ensis”. Differential diagnosis: Gs slender, with angular lateral expansions and numerous pits; anterior sensilla (LM4, 5, 13, 14) spaced closely; posterior sensilla (LM6, 8, 15, 17) forming a transverse rectangle. Cr slender. P4Exp3 and P4Enp3 wide, latter with short distal section and outer seta (LM6) inserted at the same distance from base as inner distal seta (LM3), outer apical spine longer than inner apical spine. P4CxBp with widely spaced spiniform processes (LM3, 5) and broad compared to length. Fifth leg with spinules at base of subapical spine.

Re-descriptions of *Acanthocyclops americanus* (Marsh, 1893), *A*. *robustus* (Sars, 1863), and *A*. *vernalis* (Fisher, 1853), including examined specimens and synonymy, are given in Supplementary Note. For discrimination of species analyses also see Supplementary Note and Supplementary Table S5.

## Discussion

Our analyses showed that species could be distinguished in this complex by a number of morphological characters, despite extensive variability. For details see Supplementary Note. The ease with which it was possible to distinguish them using P4Enp3 was surprising, considering that we mapped only seven LMs and that this structure was used unsuccessfully in numerous previous taxonomic studies^16,19,20,34,35,41^. However, previous studies were mostly based on the nature of armature and their relative length, not on the segment’s shape. Discriminant function analyses showed that the shape of P4Exp3 could also be used alone to correctly classify all specimens of both sexes in all species pairs even after cross-validation, except for *europensis-vernalis* where the results were still above 90%. When sexes were analysed separately, however, there was no misclassification. This structure was not used previously in taxonomy because of its variability^18,19,24,40^.

P4Exp3 and P4Enp3 also showed strong covariation, unlike other structures. The low level of covariation between P4CxBp and either P4Exp3 or P4Enp3 was surprising, as they are segments on the same leg, but contrasting patterns of variation for morphological structures measured on the same individuals is not a rare phenomenon, especially if they have contrasting functional roles and likely selective regimes^50^. We also discovered that one of the reasons Cr is unsuitable for species delimitation is that a large proportion of its variability in shape separates sexes, and this character was not considered sexually dimorphic previously. The shape of Gs, defined by its lateral outline, was used by Petkovski^34^, and then also by Kiefer^20,35^ and others, to distinguish *vernalis* from *robustus*, but this character alone could not separate sister-species. On the other hand, the distribution of dorsal cuticular organs on Gs (GsCo) separated species much better in morphospace, and it separated sister-species especially well. The suitability of these understudied micro-structures for delineation of closely related congeners, when used in LBGM, was already demonstrated for harpacticoid copepods^11,12^. Dodson *et al*.^16^ claimed that variation in pore pattern on urosome was minimal in the *vernalis*-complex and not useful. Our results contradict this.

Another surprise was the limited usability of spinules for species delineation, considering that they were often used to describe cyclopoids^48^, including *Acanthocyclops* species^36^. We demonstrated that the number of spinules largely depends on the size of specimens, and that their position provides more information than their number. This is the first study evaluating intraspecific variability of spinules in any copepod group.

Results of the LBGM analyses made it possible to review previously published records and assign them to one of the four species. Surprisingly, no published illustrations could be assigned to the newly described species. The other three species have been identified in the past with all three valid names and a variety of newly proposed names, some of them already synonymized^14,42^, others were newly synonymized here (see Supplementary Note). One potential problem in judging species identities from published drawings is that we have no means of evaluating their accuracy. Also, in older publications it is often impossible to know if the drawings were based on multiple specimens, and these species are often sympatric and sometimes even syntopic. This means that studies of their ecology need to be re-evaluated, as seasonal variation in length^24,51^ and nature of P4Enp3 armature^19^ could partly be a consequence of species succession, as recently found for some cladocerans^52^. Similarly, extreme variation in the Cr length in the cyclopoid genus *Eucyclops* Claus, 1893, attributed to seasonal variation^53^, was partly explained by recently discovered cryptic species^54^.

Our results demonstrate the existence of intraspecific variability in the ploidy in this group^32^ and also show that *in-vitro* breeding experiments^16,40^ must be interpreted with caution in taxonomic studies. This could be useful for researchers struggling with similar problems in taxonomy of related crustaceans. For example, in the marine harpacticoid genus *Tigriopus* Norman, 1869 a majority of cross-breeding experiments between closely related North American species resulted either in no offspring or substantially reduced fitness^55^, but accidental crosses have been demonstrated between some of them and some distantly related European congeners^56^.

The LBGM analyses of the *vernalis*-complex showed that the effect of locality, while significant, was minute compared to the effects of sex and species. Therefore, it is reasonable to expect that future studies of other populations will not increase the range of variability beyond the species threshold, especially when sexes are analysed separately.

Beyond a direct contribution to taxonomy and systematics, multivariate analyses of phenotypes provide a language that can be further interpreted in evolutionary, ecological, and developmental studies, all of which in turn may help us to understand, explain, characterize, and formalize diversity. For example, it seems that the pattern of greater directional asymmetry in shape than in size that we observed in all datasets with matching symmetry is quite common for biological samples^57,58^. This means that the lack of directional asymmetry in size is probably adaptive in the *vernalis*-complex, which makes a lot of sense as it is smallest in structures that are involved most directly in locomotion (P4Exp3 and P4Enp3). The effect of locality was more pronounced in size than in shape, and it is probably more a consequence of differing environmental conditions than genetics; these species are genetically homogeneous throughout their ranges, with the same haplotypes of variable markers often recorded in highly disjunct locations, and one location harbouring a variety of haplotypes^41,42^.

Allometry is a known factor contributing to integration^2^, and discrimination between groups is often improved after size correction^59^. In our datasets allometry was moderate to low, except for Cr where it accounted for nearly a third of all variability. However, even after size correction we were not able to improve species discrimination because of a high level of sexual dimorphism. It is astonishing that nobody has ever noticed this dimorphism, and part of the reason might be that it was masked by allometry. Another contributing factor could be directional asymmetry in shape, which was larger in Cr than in any other structure. Interestingly, allometry was not significant when we analysed only cuticular organs on Gs (GsCo dataset), because Gs with all landmarks did show a significant amount of allometry (nearly 12% of total variation). This was one of the reasons we decided to test modularity in this structure. Also, there is some indirect evidence that cuticular organs evolve under different constraints than do macro-morphological structures^49^. Interestingly, no evidence for modularity was found in Gs. Tests of modularity in P4CxBp were also negative. This preliminary evidence of high integration in two of the most complex rigid structures gives an interesting perspective for future evolutionary and developmental studies in this group. Strong integration within structures and weak covariation between most of them is certainly confusing and warrants further research. Tests of evolutionary integration suggested that only Gs contained significant phylogenetic signal; unsurprisingly this structure was used in the first breakthrough in morphological species delineation in this complex^34,35^. The lack of phylogenetic signal in two structures that best delineate all four species (P4Exp3 and P4Enp3) could be interpreted as a further evidence for the decoupling of phenotypic and molecular evolutionary rates in this complex, and is probably part of the explanation for the taxonomic confusion and uncertainty that followed it for over 150 years.

## Material and Methods

### Specimen collection and examination

Samples were collected from 11 freshwater habitats in five European countries (Supplementary Tables S6 and S7), using an 80 µm mesh-size plankton net. Specimens were preserved in 96% ethanol. Thirty-eight females were successfully sequenced for at least one of the four molecular markers investigated (all destroyed in the process). An additional 100 females and 47 males from six localities were sampled for LBGM. These are preserved dissected on microscope slides in Faure’s medium and deposited in the Western Australian Museum (WAM). They were examined and drawn using a drawing tube attached to a Leica MB2500 phase-interference compound light microscope (CLM). A further 29 females and 17 males from six localities were analysed with a scanning electron microscope (SEM) Hitachi S-4700, and deposited in WAM on aluminium stubs. Another 89 females and 45 males were examined without dissecting under CLM, preserved in 99.5% ethanol, and deposited in WAM.

### Molecular data collection

Total genomic DNA was extracted using E.Z.N.A.^®^ Tissue DNA Mini Kits (Peqlab, Erlangen, Germany). Fragments of mitochondrial genes Cytochrome c oxidase subunit I (COI), Cytochrome B (CytB), and 12 S ribosomal RNA (12S), and nuclear gene Internal Transcribed Spacer 1 (ITS-1) were amplified using PCR primers LCO1490/HCO2198^60^, UCYTB151-F/UCYTB270-R^61^, L13337/H13845^62^, and SP-1-5′138/SP-1-3′^63^ respectively. PCR reaction was done in a Biometra T3000 cycler. Amplification reactions and PCR protocols were as in Bláha *et al*.^41^. Annealing temperatures were 48 °C for COI, 50 °C for 12 S, 55 °C for CytB, and 60 °C for ITS-1. PCR products were purified with NucleoSpin^®^ (Macherey-Nagel, Düren, Germany) and sequenced on an ABI automatic capillary sequencer (series 373) (Macrogen, Seoul, Korea), using amplification primers.

### Molecular data analysis

Phylogenetic relationships were reconstructed using partial COI, CytB, 12S, and ITS-1 sequences; however, the COI was not obtained for *vernalis* (Supplementary Table S6), thus it was analysed separately with related sequences available from GenBank. Sequences were aligned with MAFFT v7.017^64^ implemented in GENEIOUS v8.0.5 (www.geneious.com)^65^; CytB and COI alignments were translated into amino acids to check for indels and stop codons. Analysis of synonymous and non-synonymous substitutions were done in DnaSP v5.10.01^66^ to omit usage of pseudogenes. Pairwise model-corrected genetic distances were calculated for each gene in PAUP* v4.02b^67^, for which we report the mean genetic distance in order to compare the relative amounts of divergence of each gene and among particular species. Genes 12S and ITS-1 were tested for poorly aligned regions using Gblocks Server 0.91b^68^, with default settings, and the ambiguously aligned positions detected were subsequently excluded. For each alignment a corresponding nucleotide model of evolution was applied. HKY + G model was chosen by Bayesian information criterion (BIC) estimated in jModelTest v2.1.7^69^ for COI alignment and combined dataset; HKY + G (CytB and 12S) and TrN + G (ITS-1) were chosen for alignments of particular genes. A maximum likelihood (ML) tree was constructed in RAxML v7.2.8^70^ implemented in GENEIOUS, with each partition having its own GTRGAMMA model, and nodal support of the tree was tested via 2000 bootstrap replicates. Bayesian analyses were conducted in BEAST v1.8.1^71^ using partition for particular genes and corresponding nucleotide models of evolution; analyses were run under a Yule prior^72^, with input files constructed with BEAUti; three runs were conducted with a chain length of 10 million iterations and a burn-in of 1 million; the three runs were analysed in Tracer^73^ to check for chain convergence and combined in LogCombiner; TreeAnnotator was used for the production of the phylogram.

### Morphometric data collection

A pilot comparison of LBGM data collection from SEM photographs, CLM photographs, and line drawings (based on Cr) showed that line drawings introduced the smallest amount of measurement error, so all structures were sampled this way. The urosome was dissected and positioned with the dorsal side up^12^, and LMs were mapped for Gs and Cr on a paper using a drawing tube, always under the same magnification and always twice (so that imaging error could be calculated). The fourth legs were dissected and mounted with the anterior side up, and LMs were scored in the same manner for P4CxBp, P4Exp3, and P4Enp3. Numerous asymmetries and abnormalities (Supplementary Fig. S1) made it impossible to score all structures for all specimens (Supplementary Table S7). All LMs on P4Exp3 and P4Enp3 were peripheral corners (Fig. 1C). Cr (Fig. 1A) additionally included one landmark at base of the dorsal seta (LM7), while P4CxBp (Fig. 1C) also included two pores on the anterior surface (LM12, 15) and bases of terminal spinules in two rows on the posterior surface (LM10, 11, 13, 14). Gs included the greatest number and variety of LMs: peripheral corners (LM1, 2, 9, 10, 11, 18), bases of paired dorsal sensilla (LM4–8, 13–17); one median pore (LM19), bases of setae on the sixth leg (LM3, 12), and equally-spaced sliding LMs on lateral edges (LM20–45). The choice of LMs was mostly based on minimizing the possibility of a landmark swap. Cuticular pores on P4Exp3 and P4Enp3 (Fig. 1C; Supplementary Fig. S13) were not scored because of their variability. Gs included additional pores and sensilla in some species to those 11 scored (Fig. 1A), but they were also variable, as were most cuticular organs on prosomites (Supplementary Fig. S1 should suffice as an illustration). The number of spinules was counted in two rows on P4CxBp (ε, ζ; Fig. 1C), four rows on the basis of antenna (α, β, γ, δ; Fig. 1B), and along the distal margin of labrum (La), and box-plots for all were constructed in the BoxPlotR software (http://boxplot.tyerslab.com/).

### Morphometric data analysis

Drawings of LMs were scanned, and 2D Cartesian coordinates were mapped with tpsDIG v2.17^74^. Coordinates were aligned using generalized Procrustes superimposition (GPA) in MorphoJ v1.06c^75^ and size (represented as centroid size) and shape (represented as Procrustes coordinates) were estimated for each digitisation (eight digitisations per individual for P4Enp3). In addition to five full datasets (Fig. 1), we performed analyses on two datasets with a reduced number of LMs: GsCo, containing only sensilla and pores from Gs (LM4–8, 13–17, 19), and P4Bp (representing basis of P4CxBp: LM2–8, 15). For datasets with object symmetry (Gs and GsCo) GPA was performed by separating shape variation into symmetric and asymmetric components^7^. A series of exploratory and inferential tests were conducted in MorphoJ in order to characterize patterns of size and shape variation across species detected by molecular tools, but also the effects of sex and locality were studied.

One-way nonparametric Procrustes ANOVA with a randomized permutation procedure (10,000 iterations) was used for quantifying relative amounts of variation at different levels^6,7^, such as to evaluate the amount and significance of measurement error, fluctuating asymmetry, directional asymmetry, and sexual dimorphism, as well as the effects of locality and species. Allometry, the variation in shape that is associated with the variation in size, was estimated by multivariate regression of shape onto centroid size, and most analyses were performed on both uncorrected and size-corrected data, the latter on regression residuals^2^. Regression analysis was also used for estimating the effects of size and shape on the number of spinules, and for visualising shape changes (as wireframe graphs) when shape was used as the dependent variable^76^. Principal component analysis (PCA), based on the covariance matrix of the symmetric component of shape variation averaged by individual, was used to explore and visualise the shape variation in the total dataset, such as delimitation of species and sexes in morphospace. Visualisations of shape changes at the observed extremes for each eigenvector were performed by warping outlines prepared in tpsDIG from Fig. 1 for all datasets, except for GsCo where transformation grids were used. The overall strength of association between different datasets was estimated by RV coefficient from partial least squares (PLS) analysis for data averaged by individual and pooled by species and sex, and its significance was estimated after 10,000 permutations against the null hypothesis of complete independence^77^. Warped outlines were used to visualise corresponding shape changes for PLS axes. Data integration was also estimated from the percentage of overall variation in the first two eigenvectors, and from matrix correlation between individual variation and fluctuating asymmetry, both overall and pooled by species and sexes, with and without diagonal blocks^6,7^. As some studies^12,49^ suggested that cuticular organs might have evolved under different evolutionary pressures than macro-morphological structures, we tested several modularity hypotheses in the two complex structures that contained them (Gs and P4CxBp). For Gs we could not use the entire dataset, as the sample size (100 females) did not substantially exceed the dimensionality of data (45 LMs in 2D equals to 86 degrees of freedom after Procrustes fit). Phylogenetic signal in the LBGM data was tested by plotting PCA scores (both uncorrected and size-corrected) onto the concatenated tree using squared-change parsimony and a 10,000 random permutation test that stimulates the null hypothesis of no phylogenetic signal by randomly swapping the shape data among the terminal nodes^78^. Raw data were plotted onto the concatenated tree to produce warped outlines for species means for both sexes. Discriminant function analysis (DFA) with cross-validation was used to examine the separation of individuals in all species pairs.

## Supplementary information

Supplementary Information
