# Supplementary material for: Taming extreme morphological variability through coupling of molecular phylogeny and quantitative phenotype analysis as a new avenue for taxonomy
Source: Sci Rep. 2019 Feb 20;9:2429. doi: 10.1038/s41598-019-38875-2 (PMC6382794; doi:10.1038/s41598-019-38875-2)
Supplement: Supplementary file 1 — Supplementary Information [file 41598_2019_38875_MOESM1_ESM.pdf]

Taming extreme morphological variability through coupling of molecular phylogeny and quantitative phenotype analysis as a new avenue for taxonomy

Tomislav Karanovic[1] & Martin Bláha[2]

[1]Hanyang University, Department of Life Science, Seoul 133-791, South Korea. [2]University of South Bohemia, Faculty of Fishery and Protection of Waters, South Bohemian Research Centre of Aquaculture and Biodiversity of Hydrocenoses, Zátiší 728/II, 38925 Vodňany, Czech Republic. Correspondence and requests for material should be addressed to T.K. (email: tomislav@hanyang.ac.kr)

SUPPLEMENTARY NOTE

Results

Allometry. Because of the pronounced differences in size between species, as well as between sexes within species (Table 1), it was reasonable to expect that allometry (the variation is shape that is associated with variation in size) influences the patterns of variation. To remove allometry from our datasets we regressed the symmetric component of individual means from ANOVA analyses (as dependent variable) onto centroid size of individual means (independent variable), and performed a permutation test with 10,000 rounds, pooled by species and sex (as two major integrating factors). Allometry was statistically highly significant ($P < 0.0001$) in all datasets, except for GsCo where it accounted for only 1.27% ($P = 0.2637$) in the symmetric component and 0.82% ($P = 0.5548$) in the asymmetric component of the overall shape variation. The highest allometry was found in Cr (28.64%), followed by moderate amounts in P4CxBp (12.73%) and Gs (11.95%), and small amounts in P4Exp3 (6.51%), P4Enp3 (5.61%), and P4Bp (3.24%). Therefore, all further analyses were performed on allometry-free (size-corrected) data (represented as regression residuals), as well as on uncorrected data.

Evolutionary integration. Evolutionary integration of our morphometric data was tested by plotting PCA scores (Figs. 3, 4, Supplementary Figs. S3, S4, S5) onto the concatenated tree (Fig. 2) using squared-change parsimony for all five structures with and without allometry (Supplementary Fig. S8). As expected from our regression analyses (see above), the distribution of taxa in morphospace defined by the first two eigenvectors changed more for those structures that showed more allometry (Cr and P4CxBp) than for those that showed little (P4Exp3 and P4Enp3). Randomized permutation test, which swapped the shape data among the terminal nodes for 10,000 rounds, suggested that for uncorrected data only Gs contains statistically significant phylogenetic signal ($P < 0.0001$); for all other structures the P-value exceeded 0.32 (Supplementary Fig. S8). The results were highly improved for size-corrected data, where only two structures (P4Exp3 and P4Enp3) showed statistically insignificant permutation tests ($P = 0.2097$ and $P = 0.6659$ respectively); this is interesting as these two structures are most effective for species delimitation in morphospace.

Discrimination of species. Discriminant function analysis (DFA) with cross-validation showed statistically highly significant results ($P < 0.0001$) for all seven datasets, both in parametric tests and after 10,000 permutation runs. Structures on the fourth leg (P4Exp3, P4Enp3, and P4CxBp) correctly classify all specimens for all species pairs, except for *europensis-vernalis* (Supplementary Table S5). The lowest scores were recorded for Gs for *americanus-vernalis* (65% and 74% correctly classified specimens respectively) and GsCo for *robustus-vernalis* (65% and 85%). Only one other structure showed scores of less than 80% (Gs for *robustus-vernalis*), while most structures showed scores of more than 90% on at least one side. Not surprisingly, distinguishing the new species from its phylogenetic sister-species proved most problematic (the *europensis-vernalis* pair), with no structure correctly classifying all specimens on both sides (Supplementary Table S5), but several showed very good results, including Cr (98% and 100% correctly classified specimens respectively), Gs (97% and 100%), GsCo (100% and 96%), P4Enp3 (93% and 100%), and P4Exp3 (92% and 100%). As the GsCo is the only dataset that correctly classified all specimens of *europenis* from *vernalis*, it could be suggested as an ideal morphological taxonomic character (Supplementary Fig. S6), in combination with P4Enp3 that correctly classified all specimens in all other species pairs and shows the greatest range of morphological variability (see Fig. 3).

Taxonomy. ***Acanthocyclops vernalis*** **(Fisher, 1853)** (Supplementary Figs. S11-S17).

Synonymy: *Cyclops vernalis* – Fischer[1], tab. III, figs. 1-5; Gurney[2], figs. 1598-1615; Yeatman[3], figs. 51 & 53 [not fig. 58].

Acanthocyclops vernalis – Rylov[4], fig. 47(1-4); Petkovski[5], figs. 1-9; Kiefer[6], figs. 54-99; Kiefer[7], fig. 85: Purasjoki & Viljamaa[8], fig. 3; Dodson[9], fig. 4C-G; Einsle[10], fig. 54: Alekseev *et al.*[11], fig. 3; Chang[12], fig. 243; Miracle et al.[13], fig. 1.

Acanthocyclops vernalis – Damian-Georgescu[14], fig. 67.

Acanthocyclops (s. str.) vernalis – Monchenko[15], figs. 80 & 81.

Acanthocyclops (s. str.) vernalis f. *robusta* – Monchenko[15], fig. 82(1-3) [not fig. 82(4-90].

Acanthocyclops robustus – Chang[12], fig. 246.

Cyclops lucidulus – Sars[16], plate XXV.

Cyclops americanus – Marsh[17], fig. 9 [not figs 8 & 10].

Material examined: 19 females and four males dissected on one slide each (WAM C55977-99) from Sousedovice, Czech Republic (see Table 1); eight females and nine males dissected on one slide each (WAM C56000-12) from Vodňany, Czech Republic (Table 1); three females and one male on one SEM stub (WAM C56013) from Sousedovice; five females (one dissected) and three males on one SEM stub (WAM C56014) from Vodňany; 16 females and three males in two alcohol vials (WAM C56015-6) from Vodňany; and five females in one alcohol vial (WAM C56017) from Sousedovice.

Differential diagnosis: Gs with angular lateral anterior expansions and numerous surface integumental pits; anterior sensilla (LMs 4, 5, 13, 14) spaced widely; posterior sensilla (LMs 6, 8, 15, 17) forming a trapezoid. Cr very slender. P4Exp3 and P4Enp3 very narrow, latter with short distal section and outer seta (LM 6) inserted at the similar

distance from base as inner distal seta (LM 3), and with outer apical spine longer than inner apical spine. P4CxBp with widely spaced spiniform processes (LMs 3, 5) and generally broad compared to length. Fifth leg with large spinules at base of subapical spine.

***Acanthocyclops robustus* (Sars, 1863)** (Supplementary Figs S11-16, S18).

Synonymy: *Cyclops robustus* – Sars[16], plate XXVI.

Acanthocyclops robustus – Mastrantuono & Stella[18], figs. 1-9; Petkovski[5], figs. 32-41 [not figs. 21-31]; Kiefer[7], fig. 84N [not fig. 84O, USA]; Mastrantuono[19], figs. 1-7; Morton[20], fig. 1; Einsle[10], fig. 53DR [not fig. 53OS]; Mirabdullayev & Defaye[21], figs. 1-17 [not figs. 18-23]; Alekseev *et al.*[11], figs. 2H-I, 4A, C, E; Miracle *et al.*[13], fig. 2.

Acanthocyclops (Acanthocyclops) robustus var. *armata* – Dussart[22], fig. 56.

Acanthocyclops vernalis var. *robustus* – Rylov[4], fig. 47(5-7).

Acanthocyclops vernalis robustus – Damian-Georgescu[14], fig. 68.

Cyclops vernalis – Yeatman[3], fig. 58.

Acanthocyclops vernalis – Price[23], fig. 3e, g, h [not fig. 3f].

Acanthocyclops americanus – Dussart[24], figs. 1 & 2.

Acanthocyclops brevispinosus – Dodson[9], figs. 1-3; Dahms & Fernando[25], figs. 1-7.

Acanthocyclops smithae – Reid & Suárez-Morales[26], figs. 1-3.

Acanthocyclops einslei – Mirabdullayev & Defaye[27], figs. 3-24; Bláha *et al.*[28], fig. S1A-B.

Material examined: ten females and 11 males dissected on one slide each (WAM C56018-38) from Cicenice, Czech Republic (see Table 1); ten females and nine males dissected on one slide each (WAM C56039-48) from Sousedovice, Czech Republic (Table 1); five females (one dissected) and four males on one SEM stub (WAM C56049) from Cicenice; two females on one SEM stub (WAM C56050) from Sousedovice; three females on one SEM stub (WAM C56051) from Mělka Pálenina fishpond, Czech Republic, 49°14'37.017"N 13°52'14.102"E, March 2009; five females and 14 males in two alcohol vials (WAM C56052-3) from Cicenice; and 23 females in one alcohol vial (WAM C56054) from Mělka Pálenina.

Differential diagnosis: Gs smooth, with rounded lateral anterior expansions; posterior sensilla (LMs 6, 8, 15, 17) forming a square. Cr stout. P4Exp3 and P4Enp3 narrow, the latter with short distal section and outer seta (LM 6) inserted much more distally than inner distal seta (LM 3), and with outer apical spine shorter than inner apical spine. P4CxBp with narrowly spaced spiniform processes (LMs 3, 5) and narrow compared to length. Fifth leg with no spinules at base of subapical spine.

***Acanthocyclops americanus* (Marsh, 1893)** (Supplementary Figs. S11-S16, S18).

Synonymy: *Cyclops americanus* – Marsh[17], figs. 8 & 10 [not fig. 9].

Cyclops vernalis americanus – Gurney[2], figs. 1616-1625.

Acanthocyclops americanus – Alekseev *et al.*[11], figs. 2A-G, 4B, D, F, 5C [not fig. 5A]; Miracle et al.[13], figs. 3-6.

Acanthocyclops (Acanthocyclops) americanus – Dussart[22], fig. 57.

Acanthocyclops (s. str.) americanus – Monchenko[15], figs. 83-84.

Acanthocyclops robustus – Petkovski[5], figs. 21-31 [not figs. 32-41]; Kiefer[6], figs. 1-53; Kiefer[7], fig. 84O, USA [not fig. 84N]; Purasjoki & Viljamaa[8], fig. 2; Dodson[9], fig. 4A, B, 5A-E; Einsle[10], fig. 53OS [not fig. 53DR]; Lescher-Moutoué[29], fig. 1; Caramujo & Boavida[30], fig. 4; Mirabdullayev & Defaye[21], figs. 18-23.

Acanthocyclops robustus f. *limnetica* – Petkovski[5], figs. 42-64.

Acanthocyclops (s. str.) vernalis f. *robusta* – Monchenko[15], fig. 82(4-9) [not fig. 82(1-3)].

Acanthocyclops s.f. *robustus* – Ponyi[31], taf. 1 & 2.

Acanthocyclops vernalis – Price[23], fig. 3f [not fig. 3e, g, h]; Dodson *et al.*[32], fig. 1.

Acanthocyclops s.f. *vernalis* – Ponyi[31], taf. 1 & 2.

Cyclops carolinianus – Yeatman[3], figs. 59-72.

Acanthocyclops carolinianus – Einsle[10], fig. 50.

Acanthocyslops orientalis – Einsle[10], fig. 52; Chang[12], figs. 244-245.

Acanthocyclops rebecae – Fiers *et al.*[32], figs. 2-6.

Acanthocyclops trajani – Mirabdullayev & Defaye[21], figs. 24-44.

Acanthocyclops caesariatus – Mercado-Salas et al.[33], figs. 2-4.

Acanthocyclops marceloi – Mercado-Salas et al.[33], figs. 5-7.

Acanthocyclops magistridussarti – Stoch & Bruno[34], figs. 1-5.

Acanthocyclops eduardoi – Mercado-Salas & Álvarez-Silva[35], figs. 2-5.

Material examined: 17 females and 11 males dissected on one slide each (WAM C56055-82); seven females (two dissected) and five males (one dissected) on one SEM stub (WAM C56083); 16 females and 23 males in two alcohol vials (WAM C56084-5); all from Outrata, Czech Republic (see Table 1).

Differential diagnosis: Gs smooth, with rounded lateral anterior expansions; posterior sensilla (LMs 6, 8, 15, 17) forming a longitudinal rectangle. Cr stout, with long dorsal groove. P4Exp3 and P4Enp3 narrow, the latter with long distal section and outer seta (LM 6) inserted at the same distance from base as inner distal seta (LM 3), and with outer apical spine shorter than inner apical spine. P4CxBp with narrowly spaced spiniform processes (LMs 3, 5) and generally narrow compared to length. Fifth leg with no spinules at base of subapical spine.

Discussion

Smooth integument and rounded anterior expansions on Gs, as well as stout Cr and narrow P4CxBp, distinguish at once the *robustus-americanus* sister pair from *europensis-vernalis* (the latter having Gs with numerous pits and angular anterior expansions, slender Cr, and wide P4CxBp). The easiest way to distinguish all four species is by using the P4Enp3 morphology: *robustus* is the only species with the outer element (LM6) inserted more distally than the base of distal inner seta (LM3), this segment is markedly wider in *europensis* than in either *vernalis* or *americanus*, and the latter two can be distinguished by the relative length of the distal part (much longer in *americanus*) and also by the relative length of apical spines (inner longer than outer in *americanus*)

Our synonymy includes only publications with illustrations of sufficient quality to be able to identify them to one of the four species studied here; for a much more comprehensive list of references and possible synonyms see Dussart and

Defaye[36]. We examined no type material of any of the taxa synonymized here, except in one case where the first author donated specimens of *robustus* from Montenegro that were subsequently described as a new species by Mirabdullayev and Defaye[27]. Identifying previously published drawings is not an easy job, and our synonymy has to be taken with caution. As we showed in this study, every character has a wide range of variability in all four species (Figs. 3, 4, Supplementary Figs. S3-S6), and sometimes it is difficult to identify specimens based on just one character. For example, the P4Exp3 or P4Enp3 of *vernalis* and *americanus* can be quite similar in some specimens, but other characters (such as Gs) helped us to delineate these species without any problems. As mentioned in our discussion, in many publications it is not clear if the drawings of different structures were based on one or more specimens, and these species are often sympatric and sometimes even syntopic; we found all four in the close vicinity of Sousedovice, and twice two species in the same small pond: *robustus-americanus* (Supplementary Table S6) and *robustus-vernalis* (Supplementary Table S7). For judging accuracy of published drawings, we mostly relied on our previous work on other common freshwater copepod species studied and illustrated by those taxonomists, but that was not applicable in all cases and it is highly subjective.

References

01. Fischer, S. Beiträge zur Kenntnis der in der Umgegend von St. Petersburg sich findenden Cyclopiden (Fortsetzung). *Bull. Soc. Impé. Natur. Moscou* **26,** 74–100 (1853).

02. Gurney, R. *British fresh-water Copepoda* (Ray Society 1933).

03. Yeatman, H. C. American cyclopoid copepods of the *viridis-vernalis* group, (including a description of *Cyclops carolinianus* n. sp.). *Am. Midl. Nat.* **32,** 1–90 (1944).

04. Rylov, V. M. *Cyclopoida presnykh vod. Fauna SSSR, Rakoobraznye 3(3)* (Izdatelstvo Akademii Nauk SSSR, Moscow & Leningrad, 1948).

05. Petkovski, T. K. Revision von *Acanthocyclops*-Formen der *vernalis*-Gruppe aus Jugoslawien (Crustacea, Copepoda). *Acta Mus. Mac. Sci. Nat. Skopje* **14,** 93–142 (1975).

06. Kiefer, F. Revision der *robustus-vernalis*-Gruppe der Gattung *Acanthocyclops* Kiefer (Crustacea, Copepoda) (mit eingehender Beurteilung des '*Cyclops americanus* Marsh, 1892'). *Beitr. Naturkun. Forsch. Südwestdeutschl.* **35,** 95–110 (1976).

07. Kiefer, F. Freilebende Copepoda. *Das Zooplankton der Binnengewässer, Binnengewässer 2. Teil 26(2)* (eds. Kiefer, F. & Fryer, D.) 1–380 (Nägele und Obermiller, 1978).

08. Purasjoki, K. & Viljamaa, H. *Acanthocyclops robustus* (Copepoda, Cyclopoida) in plankton of the Helsinki Sea area, and a morphological comparison between *A. robustus* and *A. vernalis*. *Finn. Mar. Res.* **250,** 33–44 (1984).

09. Dodson, S. Morphological analysis of Winsconsin (U.S.A.) species of the *Acanthocyclops vernalis* group (Copepoda: Cyclopoida). *J. Crust. Biol.* **14,** 113–131 (1994).

10. Einsle, U. *Copepoda: Cyclopoida Genera* Cyclops*,* Megacyclops*,* Acanthocyclops*; Guides to the Identification of the Microinvertebrates of the Continental Waters of the World 10* (SPB Academic Publishing, 1996).

11. Alekseev, V., Fefilova, E. & Dumont, H. J. Some noteworthy free-living copepods from surface freshwater in Belgium. *Belg. J. Zool.* **132,** 133–139 (2002).

12. Chang, C. Y. *Inland-water Copepoda, Illustrated encyclopedia of Fauna and Flora of Korea, vol 42* (Ministry of Education, Seoul, 2009). [in Korean]
13. Miracle, M. R., Alekseev, V., Monchenko, V., Setandreu, V. & Vicente, E. Molecular-genetic-based contribution to the taxonomy of the *Acanthocyclops robustus* group. *J. Nat. Hist.* **47,** 5–12 (2013).
14. Damian-Georgescu, A. Copepoda. Fam. Cyclopidae (forme de apa dulce). *Fauna Republ. Pop. Rom. Crustac.* **4(6),** 1–205 (1963).
15. Monchenko, V. I. *Shchelepnoroti tsiklopodiny, tsiklopi (Cyclopidae), Fauna Ukrainy 27(3)* (Vidavnitstvo Naukova Dumka Kiev 1974).
16. Sars, G. O. Copepoda Cyclopoida. Parts III & IV. Cyclopidae (continued). *An Account of the Crustacea of Norway, with short descriptions and figures of all the species, Bergen Museum, Bergen* **6,** 33–56 (1913).
17. Marsh, C. D. On the Cyclopidae and Calanidae of central Wisconsin. *Trans. Wisconsin Acad. Sci. Arts Lett.* **9,** 189–224 (1893).
18. Mastrantuono, L. & Stella, E. Morfologia e posizione sistematica di *Acanthocyclops robustus* Sars (Crustacea Copepoda) di uno stagno del Lazio. *Riv. Idrobiol.* **13,** 211–224 (1974).
19. Mastrantuono, L. Morphological characteristics of *Acanthocyclops robustus* Sars (Crustacea Copepoda) from Lake Monterosi (Latium). *Bollet. Zool.* **47,** 39–45 (1980).
20. Morton, D. W. Revision of the Australian Cyclopidae (Copepoda: Cyclopoida). I. *Acanthocyclops* Kiefer, *Diacyclops* Kiefer and *Australocyclops*, gen. nov. *Austr. J. Mar. Freshw. Res.* **36,** 615–634 (1985).
21. Mirabdullayev, I. M. & Defaye, D. On the taxonomy of the *Acanthocyclops robustus* species complex (Copepoda, Cyclopidae), 1. *Acanthocyclops robustus* (G.O. Sars, 1863) and *Acanthocyclops trajani* n. sp. *Selevinia* **1-4,** 7–19 (2002).
22. Dussart, B. *Les Copépodes des Eaux Continentales d'Europe Occidentale, Tome II: Cyclopoïdes et Biologie* (N. Boubée & Cie, 1969).
23. Price, J. L. Cryptic speciation in the *vernalis* group of Cyclopidae. *Can. J. Zool.* **36,** 285–303 (1958).
24. Dussart, B. H. *Acanthocyclops americanus* en France (Crusacé, Copépode). *Bull. Mus. Nat. Hist. Natur. 2e Sér.* **42,** 725–729 (1971).
25. Dahms, H.-U. & Fernando, C. H. Redescription of *Acanthocyclops brevispinosus* (Herrick, 1884) (Copepoda, Cyclopoida) from Ontario. *Crustaceana* **70,** 129–144 (1997).
26. Reid, J.W. & Suárez-Morales, E. A new, neotropical species of *Acanthocyclops* (Copepoda: Cyclopoida: Cyclopoidae). *Beaufortia* **49:** 37–45 (1999).
27. Mirabdullayev, I. M. & Defaye, D. On the taxonomy of the *Acanthocyclops robustus* species-complex (Copepoda, Cyclopidae): *Acanthocyclops brevispinosus* and *A. einslei* sp. n. *Vest. Zool.* **38,** 27–37 (2004).
28. Bláha, M., Hulák, M., Slouková, J. & Těšitel, J. Molecular and morphological patterns across *Acanthocyclops vernalis-robustus* species complex (Copepoda, Cyclopoida). *Zool. Scripta* **39,** 259–268 (2010).
29. Lescher-Moutoué, F. Seasonal variations in size and morphology of *Acanthocyclops robustus* (Copepoda Cyclopoida). *J. Plankt. Res.* **18,** 907–922 (1996).

30. Caramujo, M. J. & Boavida, M. J. *Acanthocyclops robustus* external morphology: How many morphs? *Verhandl. Intern. Vereinig. Theor. Angew. Limn.* **26,** 1904–1912 (1998).

31. Ponyi, J. E. Studien über das Crustaceen-Plankton des Balaton. III. Beiträge zur Systematik und Cyclomorphose von *Acanthocyclops vernalis* (Fischer), 1853. *Ann. Biol. Tihany* **34,** 163–177 (1967).

32. Dodson, S. I., Grishanin, A. K., Gross, K. & Wyngaard, G. A. Morphological analysis of some cryptic species in the *Acanthocyclops vernalis* species complex from North America. *Hydrobiologia* **500:** 131–143 (2003).

32. Fiers, F., Ghenne, V., Suárez-Morales, E. New species of continental cyclopoid copepods (Crustacea, Cyclopoida) from the Yucatán peninsula, Mexico. *Stud. Neotr. Fauna Envir.* **35,** 209–251 (2000).

33. Mercado-Salas, N. F., Suárez-Morales, E. & Silva-Briano, M. Two new species of *Acanthocyclops* Kiefer, 1927 (Copepoda: Cyclopoida: Cyclopinae) with pilose caudal rami from semiarid areas of Mexico. *Zool. Stud.* **48,** 380–393 (2009).

34. Stoch, F. & Bruno, M. C. *Acanthocyclops magistridussarti* sp. nov., from ground waters of peninsular Italy, with comments on the intraspecific variability of the antennary basis ornamentation (Copepoda, Cyclopoida, Cyclopidae). *Crustaceana Monographs 16, Studies on Freshwater Copepoda: a Volume in Honour of Bernard Dussart* (eds. Defaye, D., Suárez-Morales, E. & von Vaupel Klein J. C.) 489–506 (Brill, 2011).

35. Mercado-Salas, N. F. & Álvarez-Silva, C. A new *Acanthocyclops* Kiefer, 1927 (Cyclopoida: Cyclopinae) from an ecological reserve in Mexico City. *J. Nat. Hist.* **47,** 5–12 (2013).

36. Dussart, B. & Defaye, D. *World Directory of Crustacea Copepoda of Inland Waters. II - Cyclopiformes* (Backhuys Publishers, 2006).

SUPPLEMENTARY FIGURES:

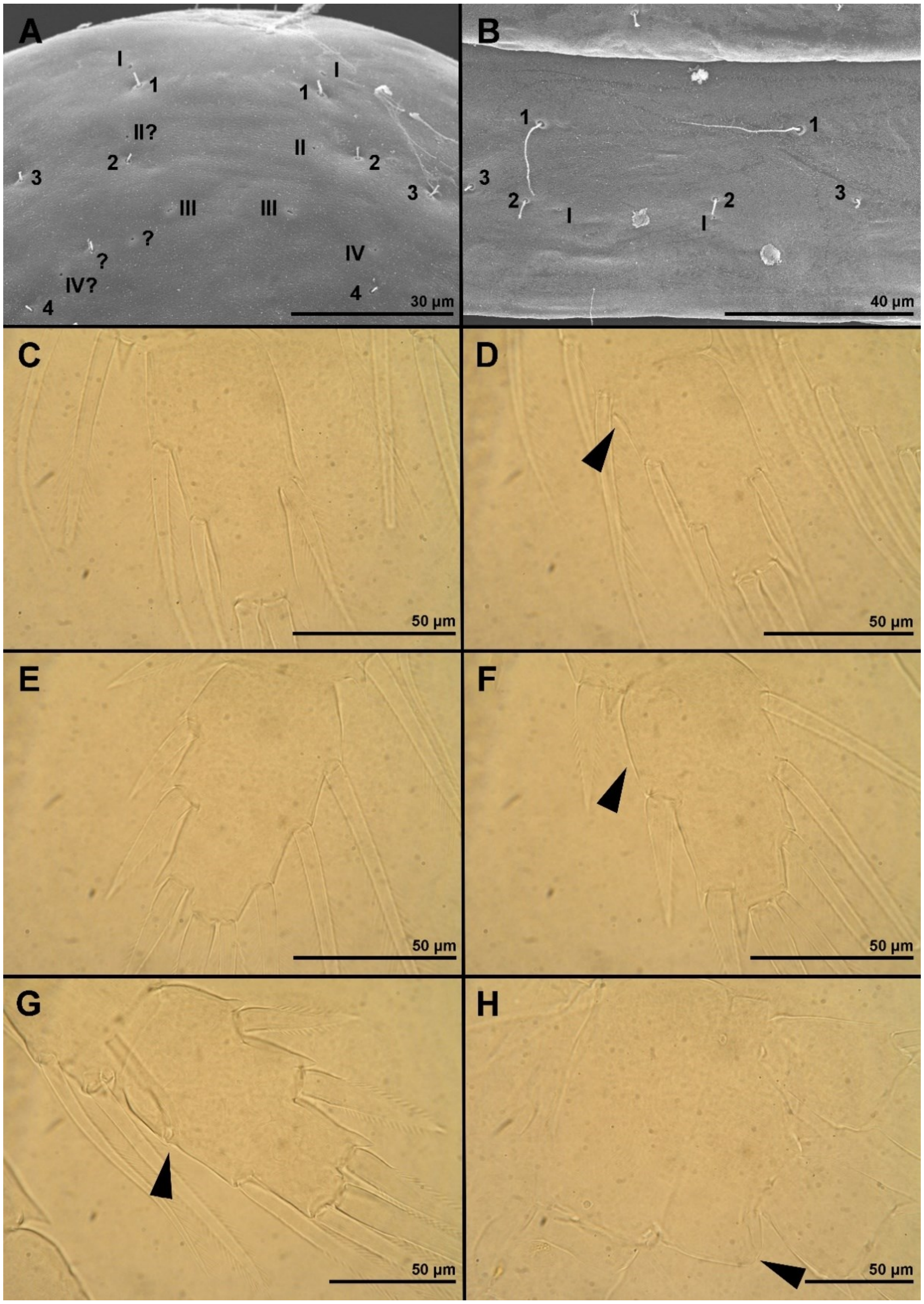

Supplementary Figure S1. Examples of extreme variability, asymmetries, and abnormalities in the *vernalis*-complex. (**A**) *A. americanus*, female from Outrata, anterior dorsal part of cephalothorax, asymmetries and uncertainties (question marks) about lateral homology of cuticular sensilla (Arabic numerals) and pores (Roman numerals); (**B**) *A. americanus*, male from Outrata, dorsal part of second pedigerous (first free) prosomite, asymmetries in distribution of cuticular sensilla and pores; (**C**) *A. europensis*, paratype female 5 from Sousedovice, normal third endopodal segment of the fourth leg (P4Enp3); (**D**) *A. europensis*, paratype female 13 from Sousedovice, deformed P4Enp3 (additional spiniform process arrowed); (**E**) *A. europensis*, paratype female 5 from Sousedovice, normal third exopodal segment of the fourth leg (P4Exp3); (**F**) *A. europensis*, paratype female 25 from Sousedovice, P4Exp3 with only two lateral spines (missing proximal spine arrowed); (**G**) *A. robustus*, female from Sousedovice, P4Exp3 with only two inner setae and with proximal seta incompletely developed (arrowed); (**H**) *A. vernalis*, male from Vodňany, basis of the fourth leg with rounded inner cuticular process (arrowed).

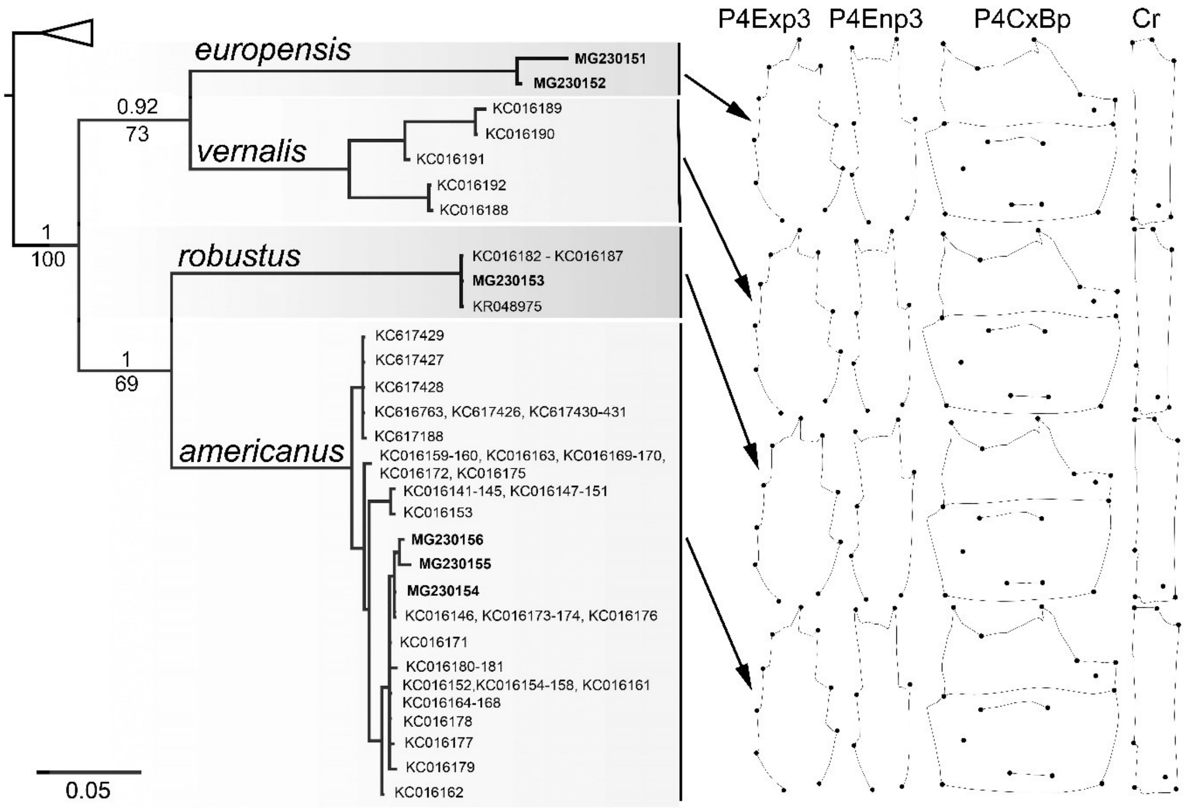

Supplementary Figure S2. Bayesian inference (BI) tree resulting from the COI dataset, depicting relationships of the four *Acanthocyclops* species studied, and warped outlines of four male morphological structures. The outlines have been scaled to the same centroid size and represent size-uncorrected mean shapes based on digitized landmarks (Fig. 1). Maximum likelihood (ML) bootstrap values and BI posterior probabilities are displayed below and above branches respectively. Specimen codes are GenBank accession numbers, and those sequenced in this study are in bold (Supplementary Table S6).

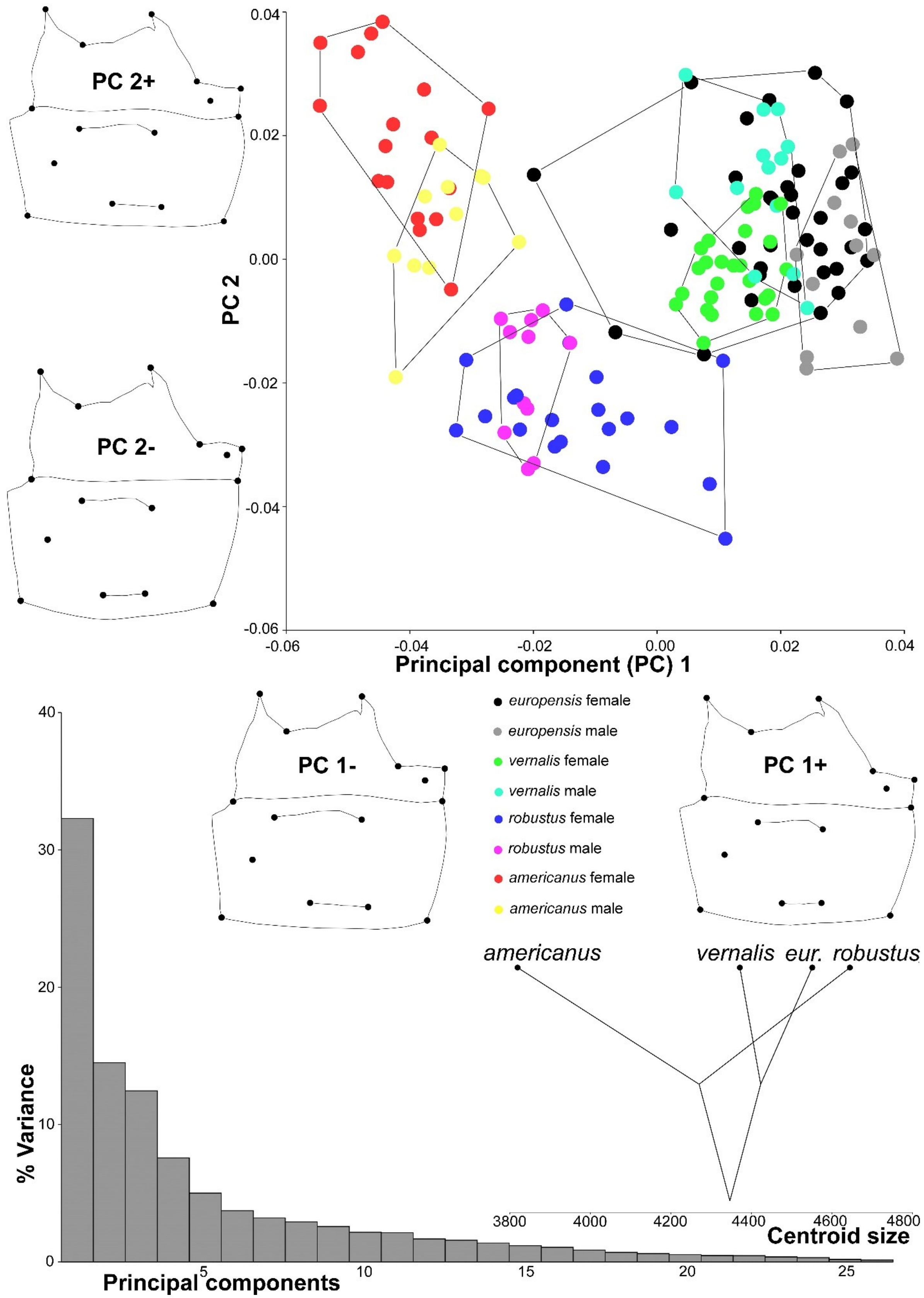

Supplementary Figure S3. Graphical visualisation of the principal component analysis based on 15 LMs in size-corrected P4CxBp dataset (Fig. 1) for 146 specimens from seven localities (Supplementary Table S7). Scatter plot shows delimitation of species and sexes as convex hulls in morphospace defined by first two eigenvectors (PCs). Warped outlines show shape changes at the observed extremes. Graph shows percentages of the total variance for each PC. Cladogram is the projection of phylogeny (Fig. 2) onto centroid size for each species mean value.

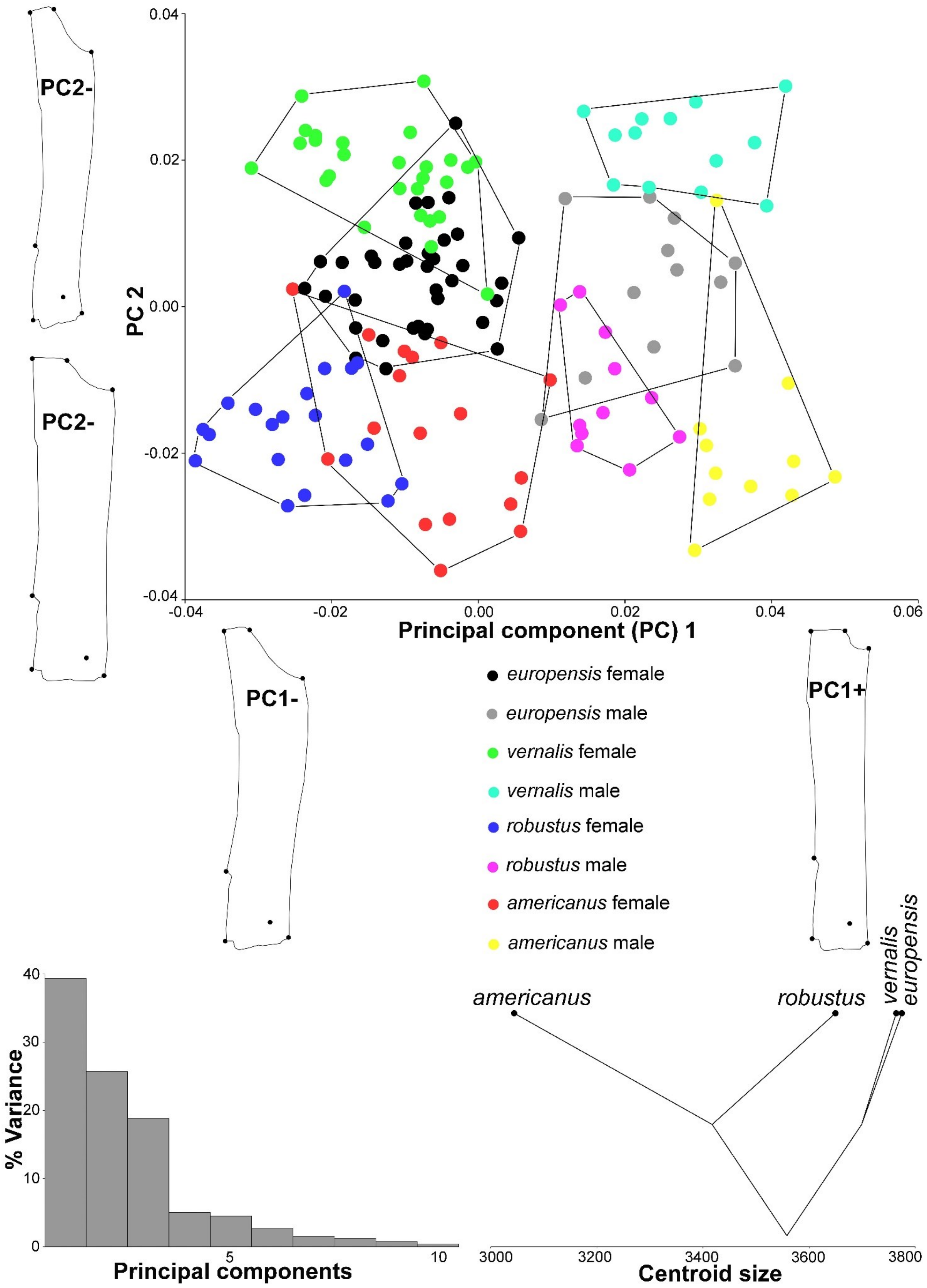

Supplementary Figure S4. Graphical visualisation of the principal component analysis based on seven LMs in size-corrected Cr dataset for 147 specimens from seven localities. Details as in Supplementary Figure S3.

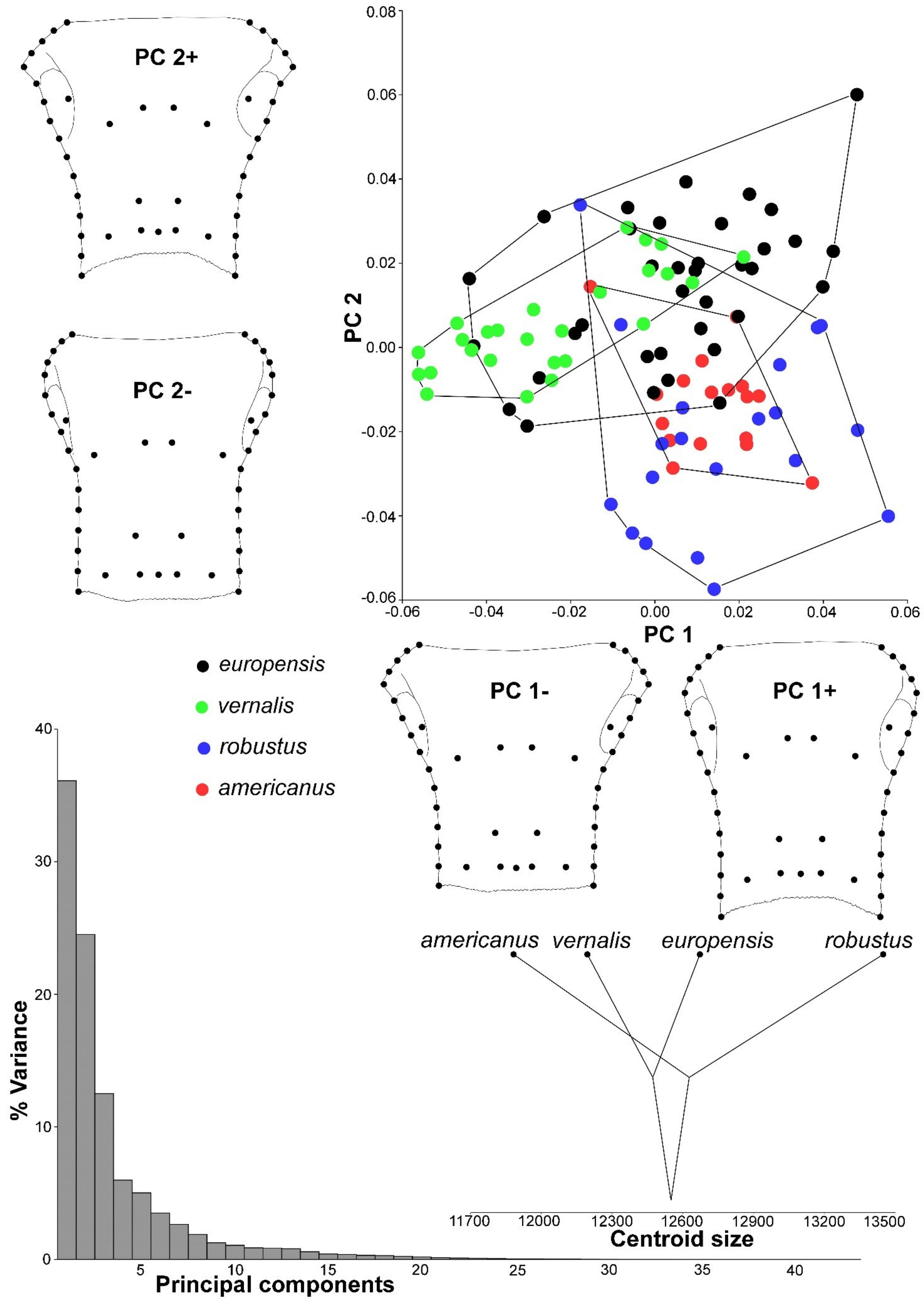

Supplementary Figure S5. Graphical visualisation of the principal component analysis based on 45 LMs in size-corrected Gs dataset for 100 females from seven localities. Scatter plot shows delimitation of species; other details as in Supplementary Figure S2.

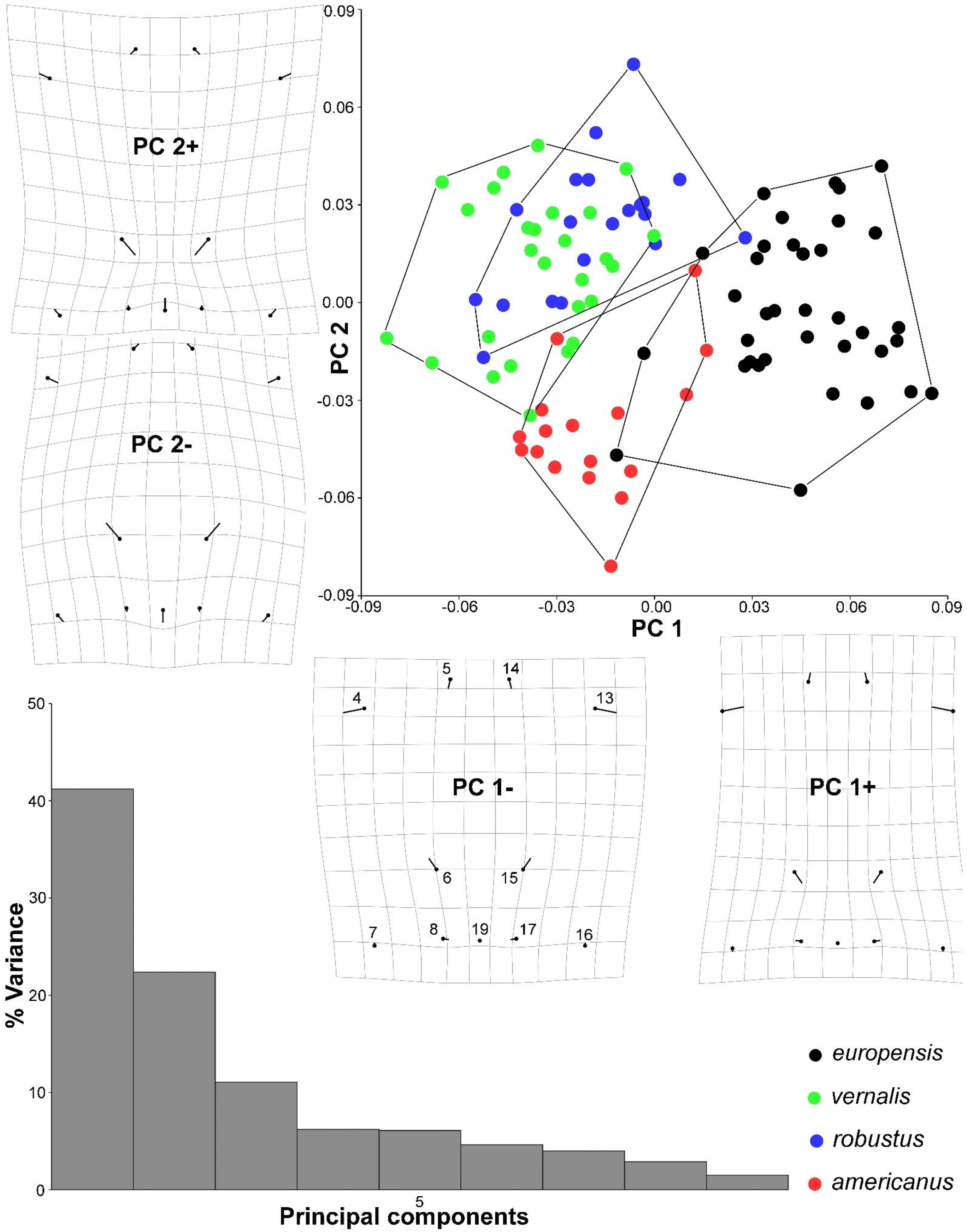

Supplementary Figure S6. Graphical visualisation of the principal component analysis for 100 females as in Figure 5, but based only on cuticular organs (GsCo dataset, size-corrected). Shape changes at the observed extremes are represented as transformation grids; other details as in Supplementary Figure S2.

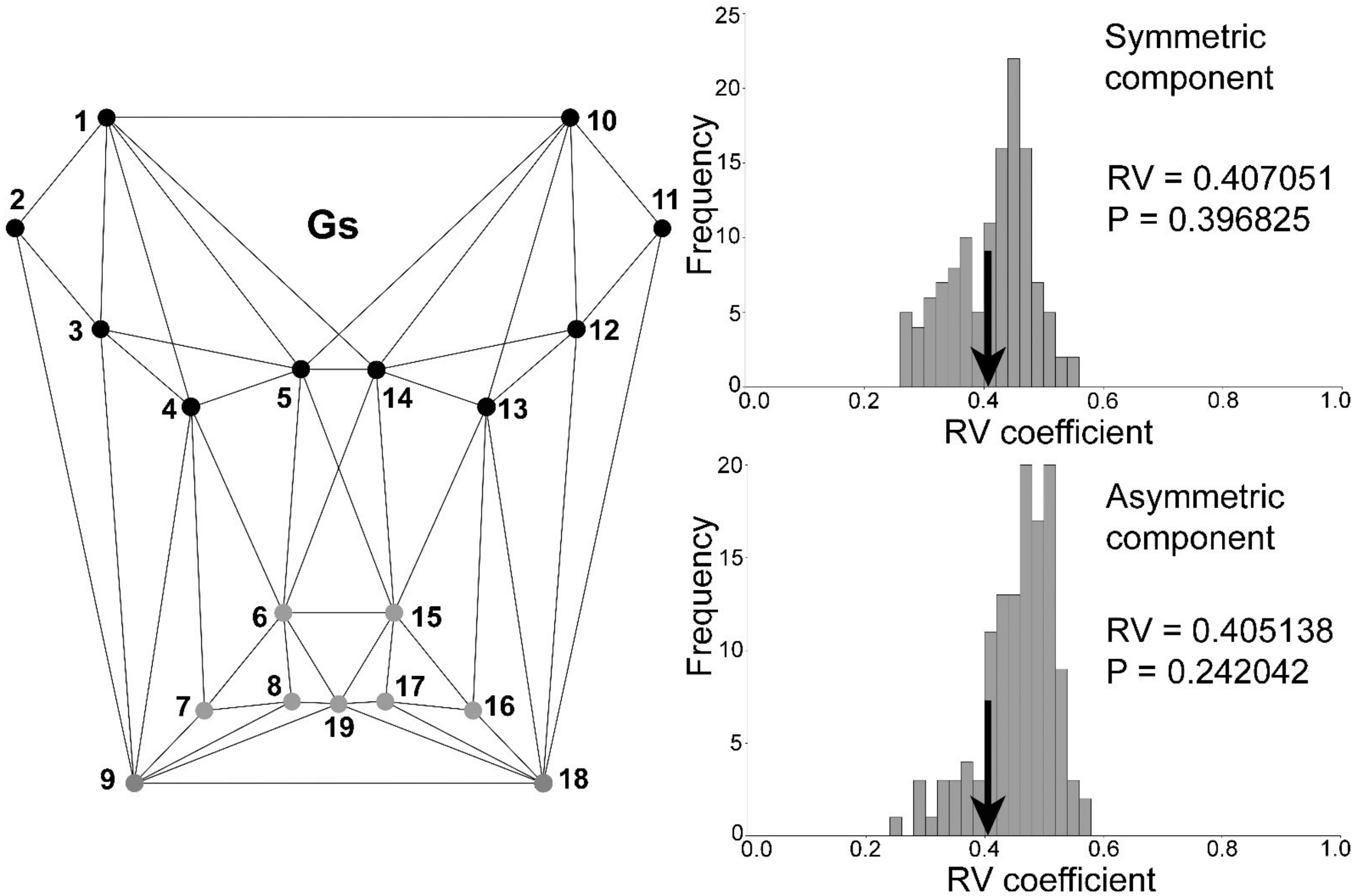

Supplementary Figure S7. Modularity hypothesis tested for a selection of LMs in the Gs dataset (see Fig. 1). Adjacency graph shows partition of landmarks into anterior (black dots) and posterior (grey dots) subsets, which correspond to the ancestral second and third urosomites respectively. Graphs show the RV coefficients (arrows) for the distribution of 10,000 alternative contiguous and non-contiguous partitions of LMs (histograms), both for the symmetric and asymmetric component of variation. P-value is estimated against the null hypothesis of total independence by randomly reshuffling observations separately within the blocks of landmark coordinates, with a new Procrustes superimposition after each permutation procedure.

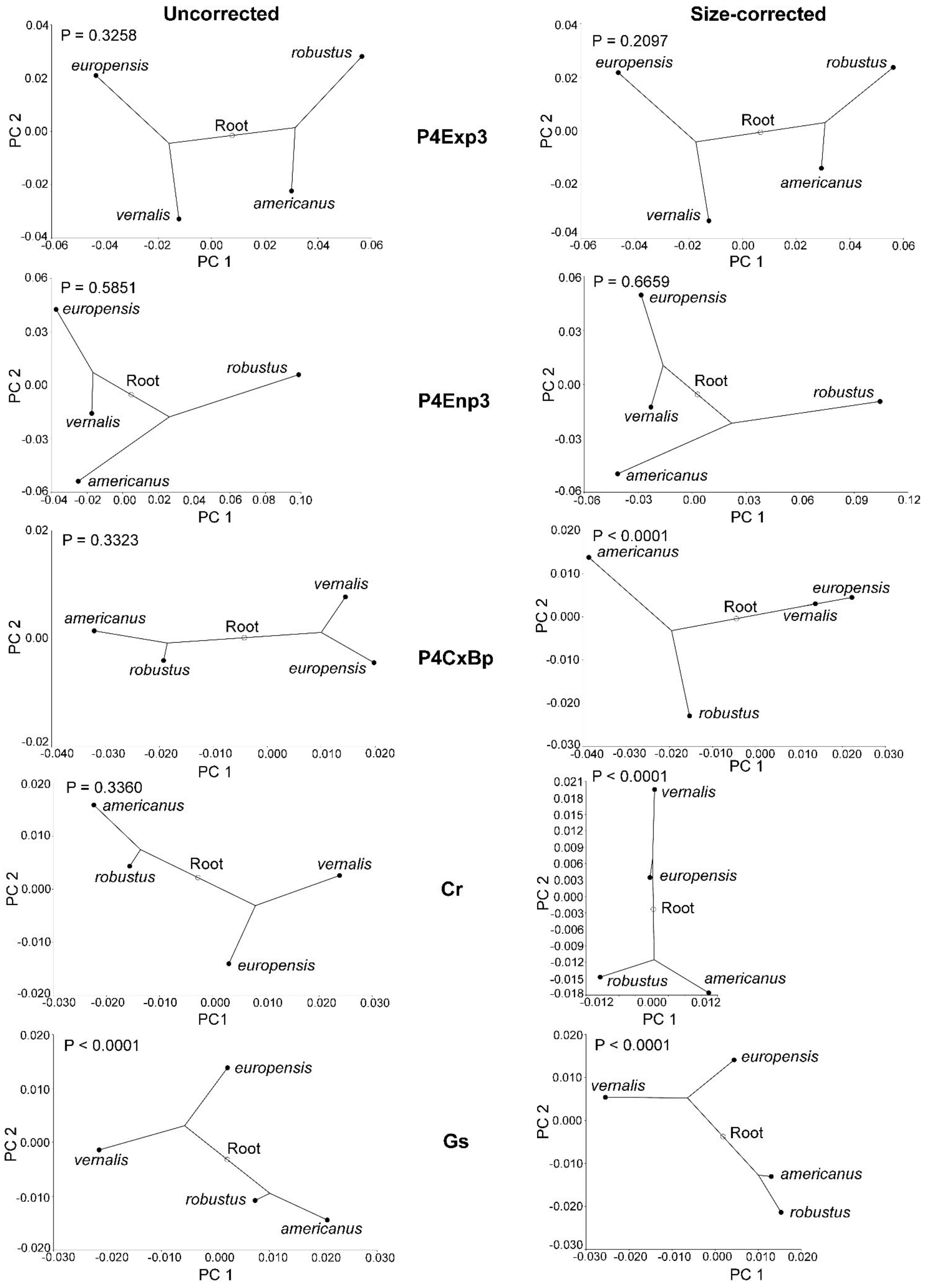

Supplementary Figure S8. Projection of the phylogeny (Fig. 2) onto morphospace, as represented by first two eigenvectors (PCs), for all five morphological structures (Fig. 1), with allometry (left) and without allometry (right). P-value is estimated after 10,000 permutation rounds by randomly exchanging the shapes among terminal nodes.

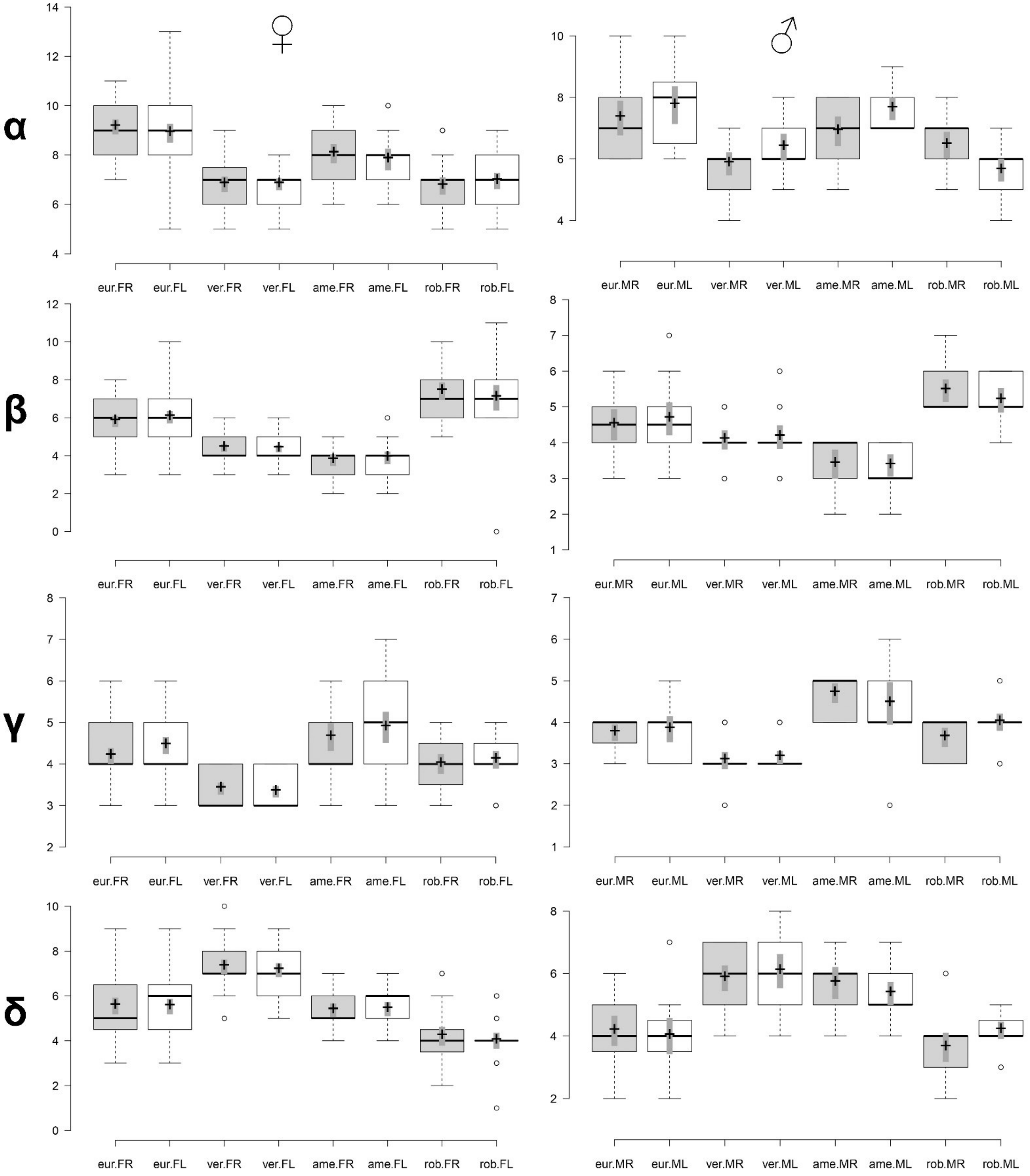

Supplementary Figure S9. Box plots showing variation in the number of spinules in four rows (Greek letters) on the basis of antenna (Fig. 1B) in females (left) and males (right). Values are given separately for the right (grey) and left side (white) in each of the four species (abbreviated by the first three letters); centre lines show the medians; box limits indicate the 25th and 75th percentiles as determined by R software; whiskers extend 1.5 times the interquartile range from the 25th and 75th percentiles, outliers are represented by dots; crosses represent sample means; bars indicate 83% confidence intervals of the means.

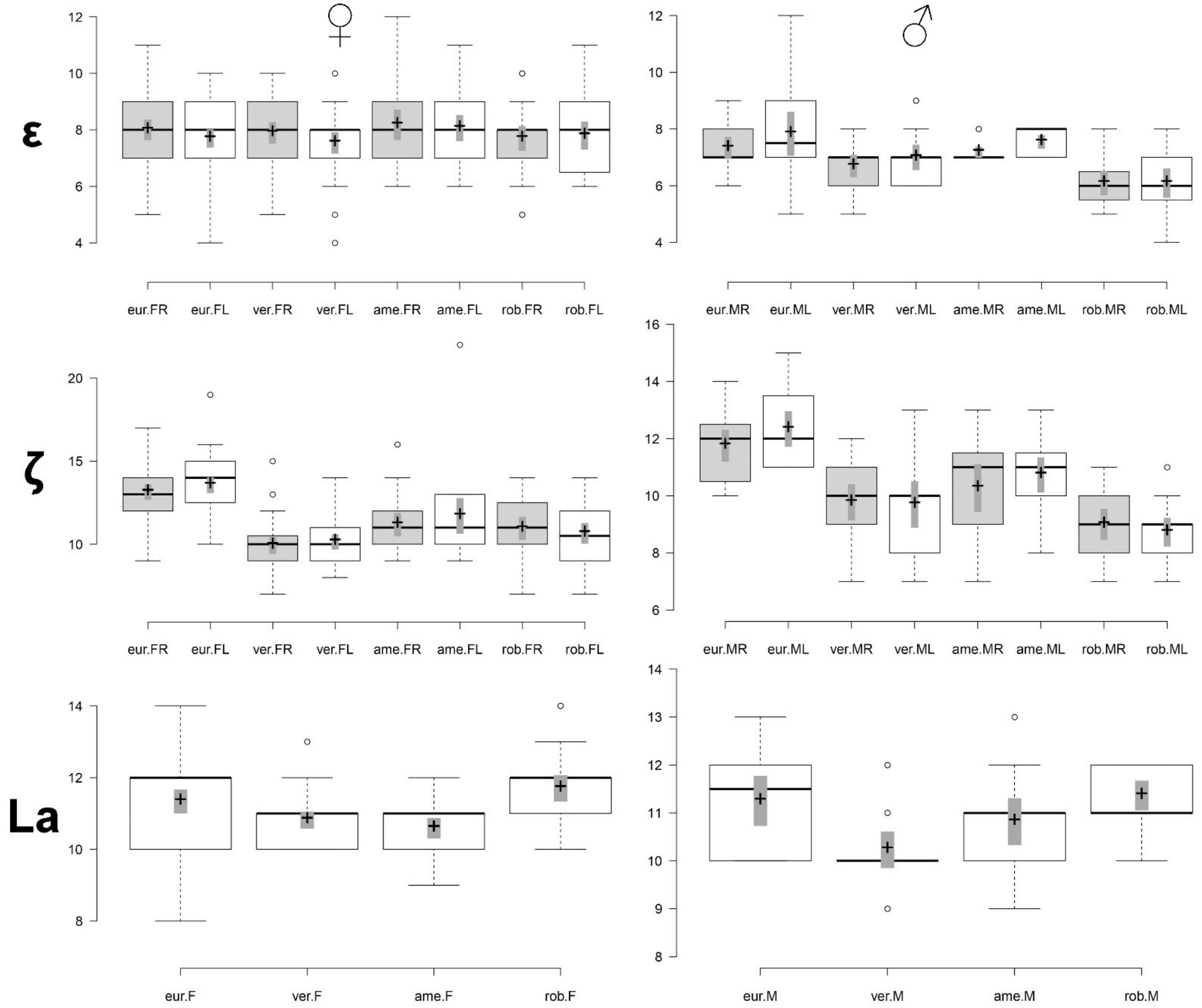

Supplementary Figure S10. Box plots showing variation in the number of spinules in two rows (Greek letters) on the coxa of fourth leg (Fig. 1C) and number of distal teeth on the labrum (La) (see Supplementary Fig. S16) in females (left) and males (right). Details as in Supplementary Figure S9, except that La has object symmetry.

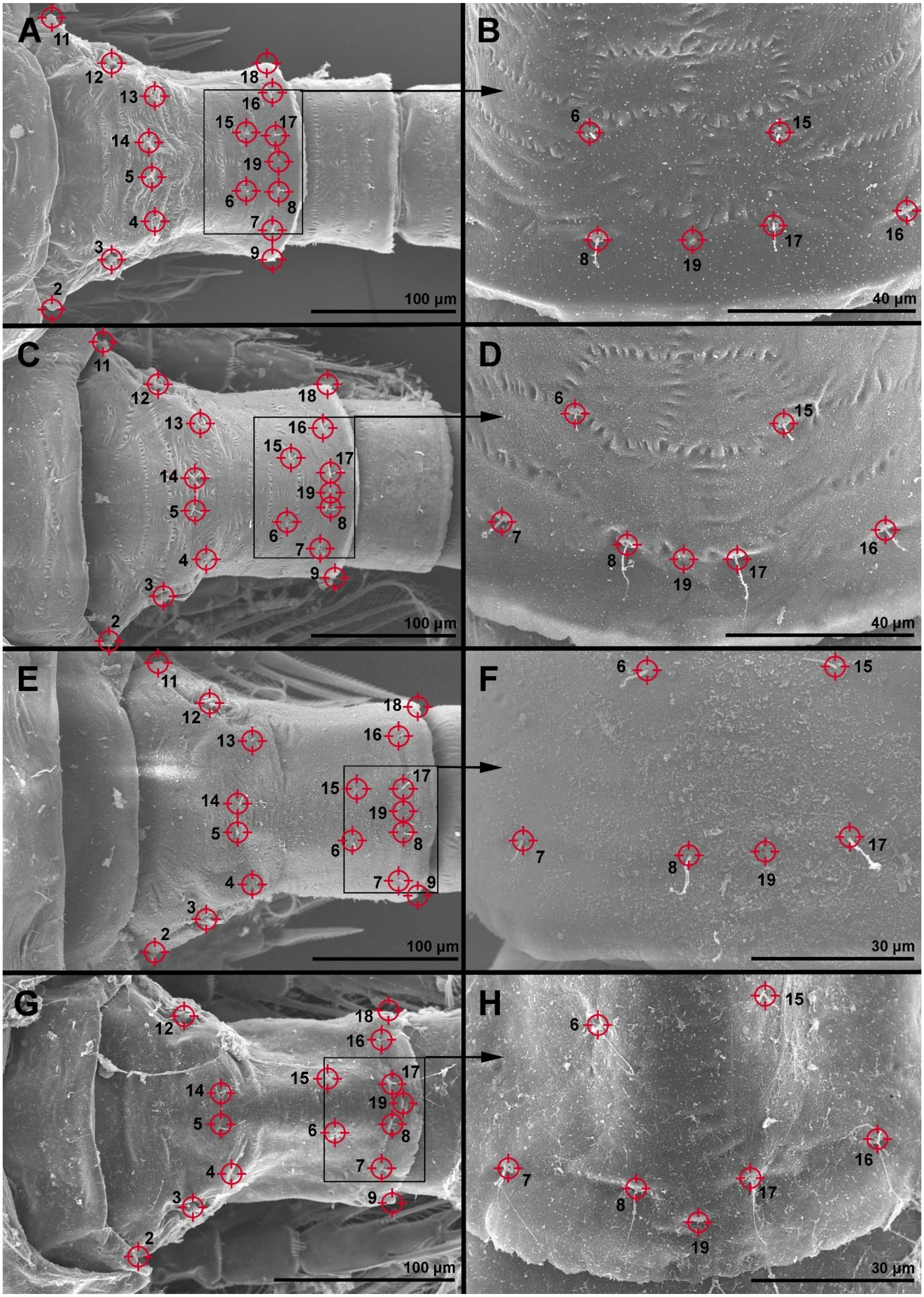

Supplementary Figure S11. Scanning electron microscope (SEM) photographs of the female Gs (left) in dorsal view and its posterior detail (right), with selected LMs marked as in Fig. 1. (**A & B**) *Acanthocyclops europensis*, Sousedovice; (**C & D**) *A. vernalis,* Vodňany; (**E & F**) *A. robustus,* Cicenice; (**G & H**) *A. americanus*, Outrata.

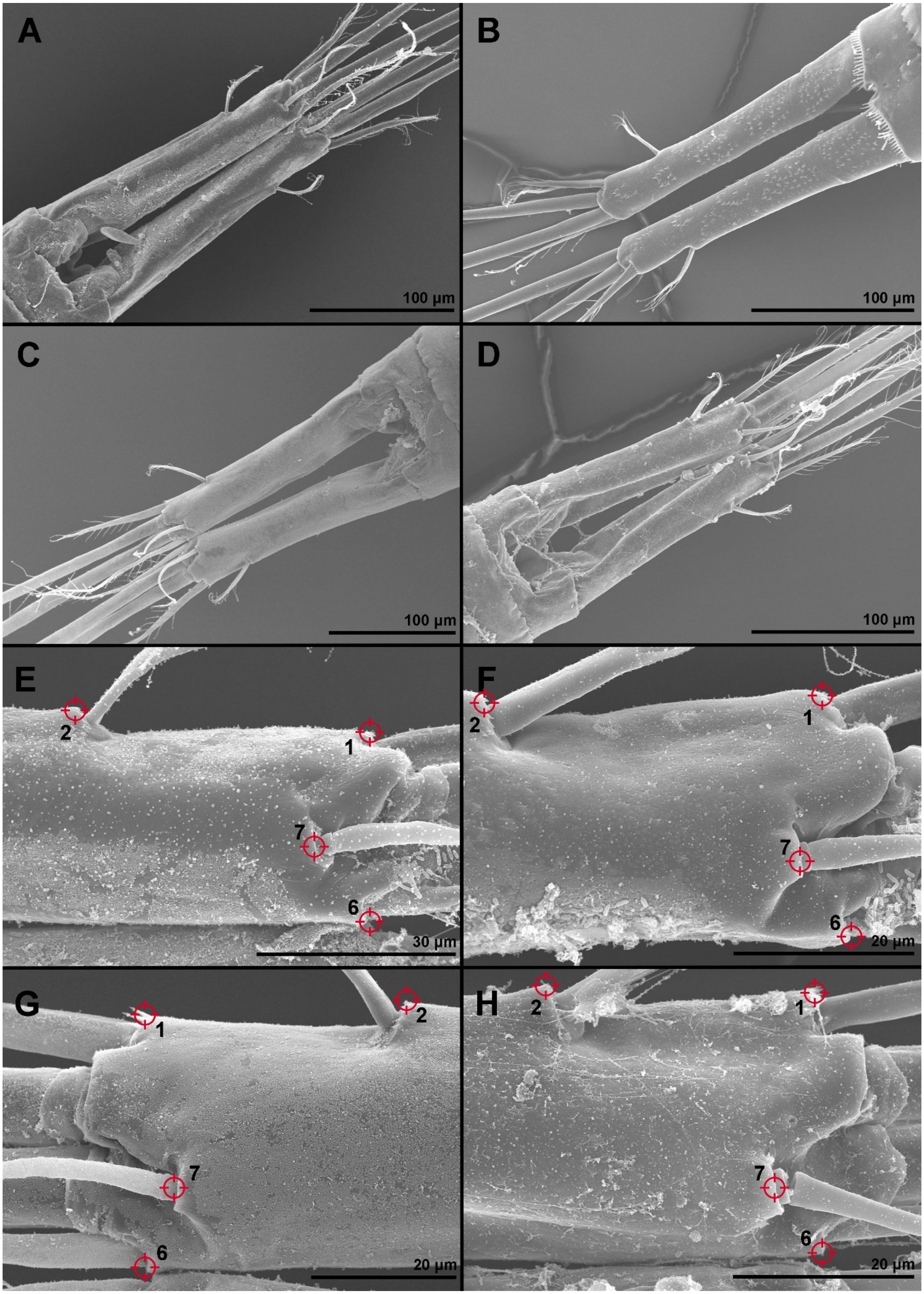

Supplementary Figure S12. SEM photographs of the female Cr (A-D) and its posterior part enlarged (E-H), all except B in dorsal view, with some LMs marked as in Fig. 1. (**A & E**) *Acanthocyclops europensis*, Sousedovice; (**B & F**) *A. vernalis,* Vodňany; (**C & G**) *A. robustus,* Cicenice; (**D & H**) *A. americanus*, Outrata.

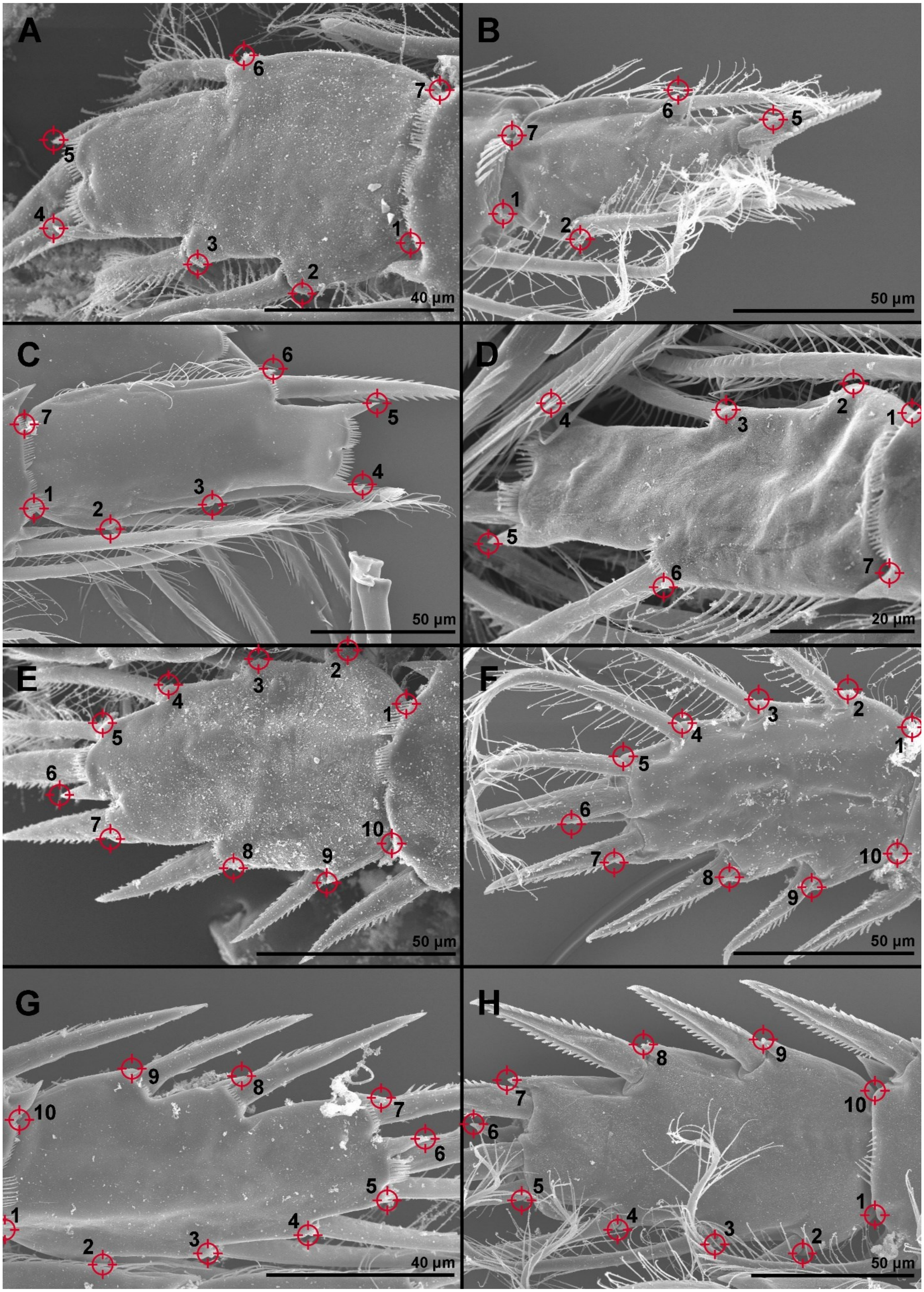

Supplementary Figure S13. SEM photographs of the female Enp3P4 (A-D) and Exp3P4 (E-H), with some LMs marked as in Fig., some in anterior view (A, C-E, G), some in posterior view (B, F, H). (**A, E & F**) *Acanthocyclops europensis*, Sousedovice; (**B & G**) *A. vernalis,* Sousedovice; (**C**) *A. robustus,* Palenina; (**D**) *A. americanus*, Outrata; (**H**) *A. robustus,* Cicenice.

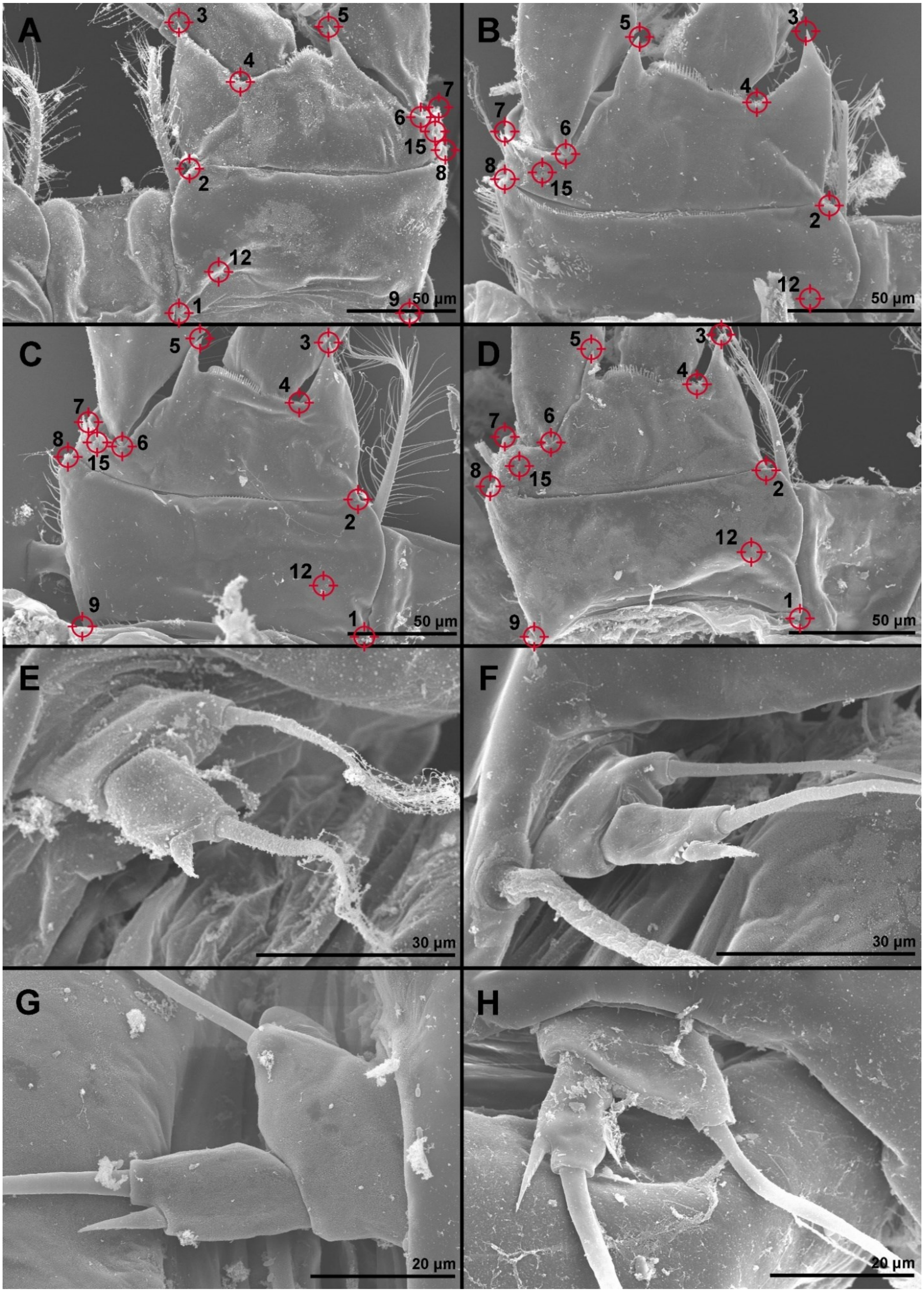

Supplementary Figure S14. SEM photographs of the female P4CxBp (A-D) and fifth leg (E-H), all in anterior view, with some LMs marked as in Fig. 1. (**A & E**), *Acanthocyclops europensis*, Sousedovice; (**B**) *A. vernalis,* Sousedovice; (**C**) *A. robustus,* Palenina; (**D & H**) *A. americanus*, Outrata; (**F**) *A. vernalis,* Vodňany; (**G**) *A. robustus,* Cicenice.

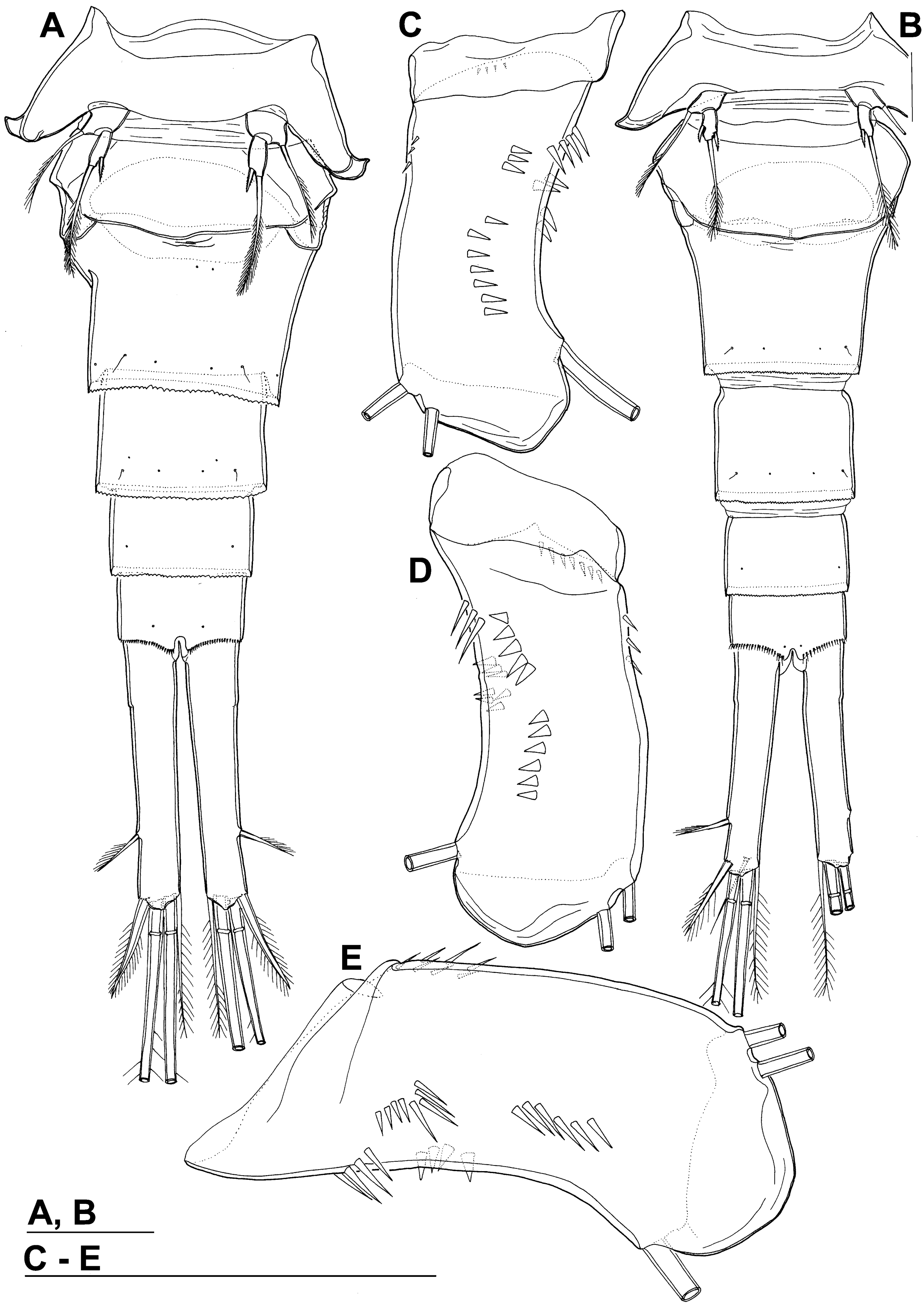

Supplementary Figure S15. Line drawings of the female urosome in ventral view (A & B) and basis of antenna in anterior view (C – E). (**A**) *Acanthocyclops europensis*, Sousedovice; (**B & D**) *A. vernalis*, Sousedovice; (**C**) *A. americanus*, Outrata; (**E**) *A. robustus*, Cicenice. Scales 100 µm.

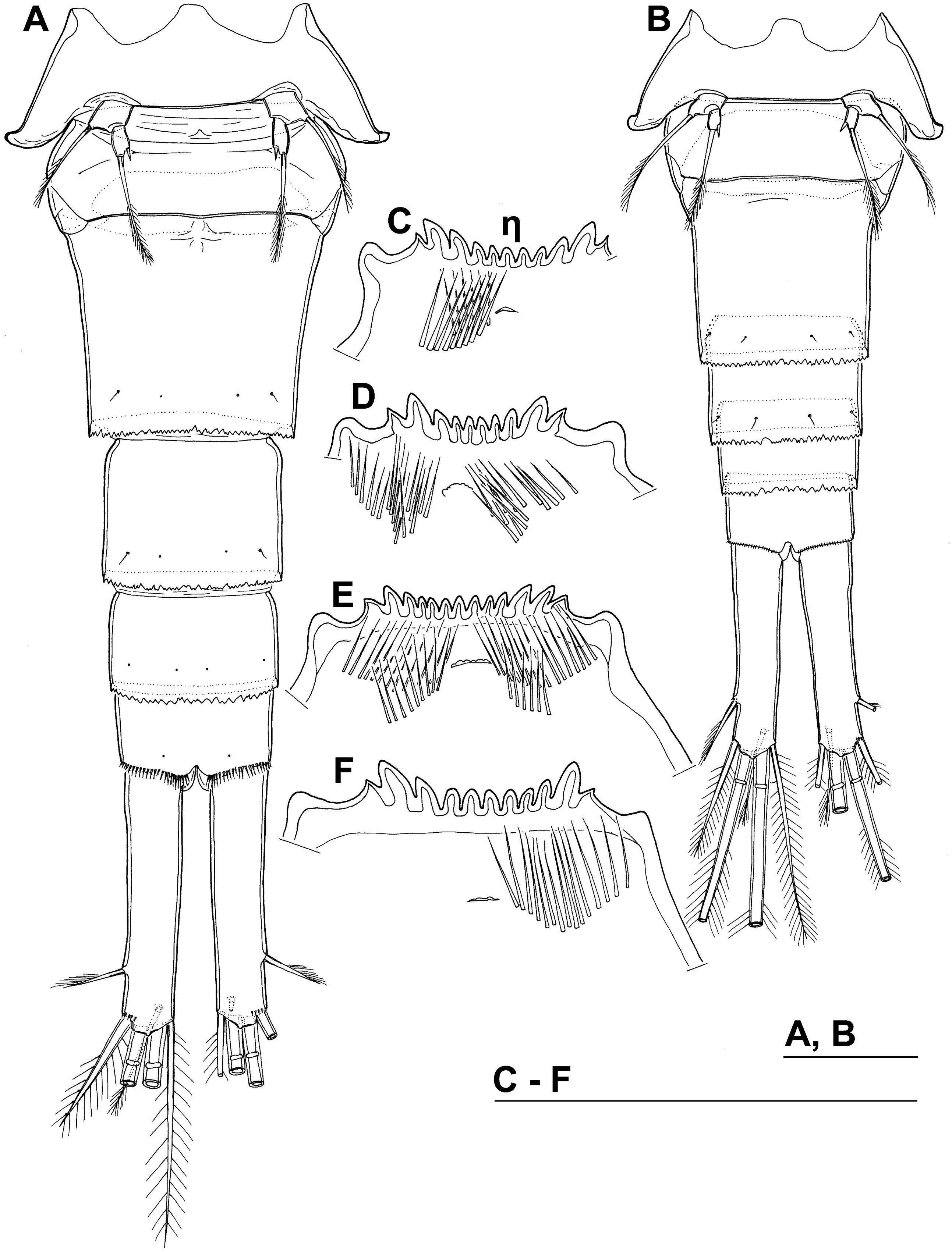

Supplementary Figure S16. Line drawings of the female urosome in ventral view (A & B) and distal tip of labrum in posterior view (C – F). (**A & F**) *Acanthocyclops robustus*, Cicenice; (**B & C**) *A. americanus*, Outrata; (**D**) *A. europensis*, Sousedovice; (**E**) *A. vernalis*, Sousedovice. Scales 100 µm.

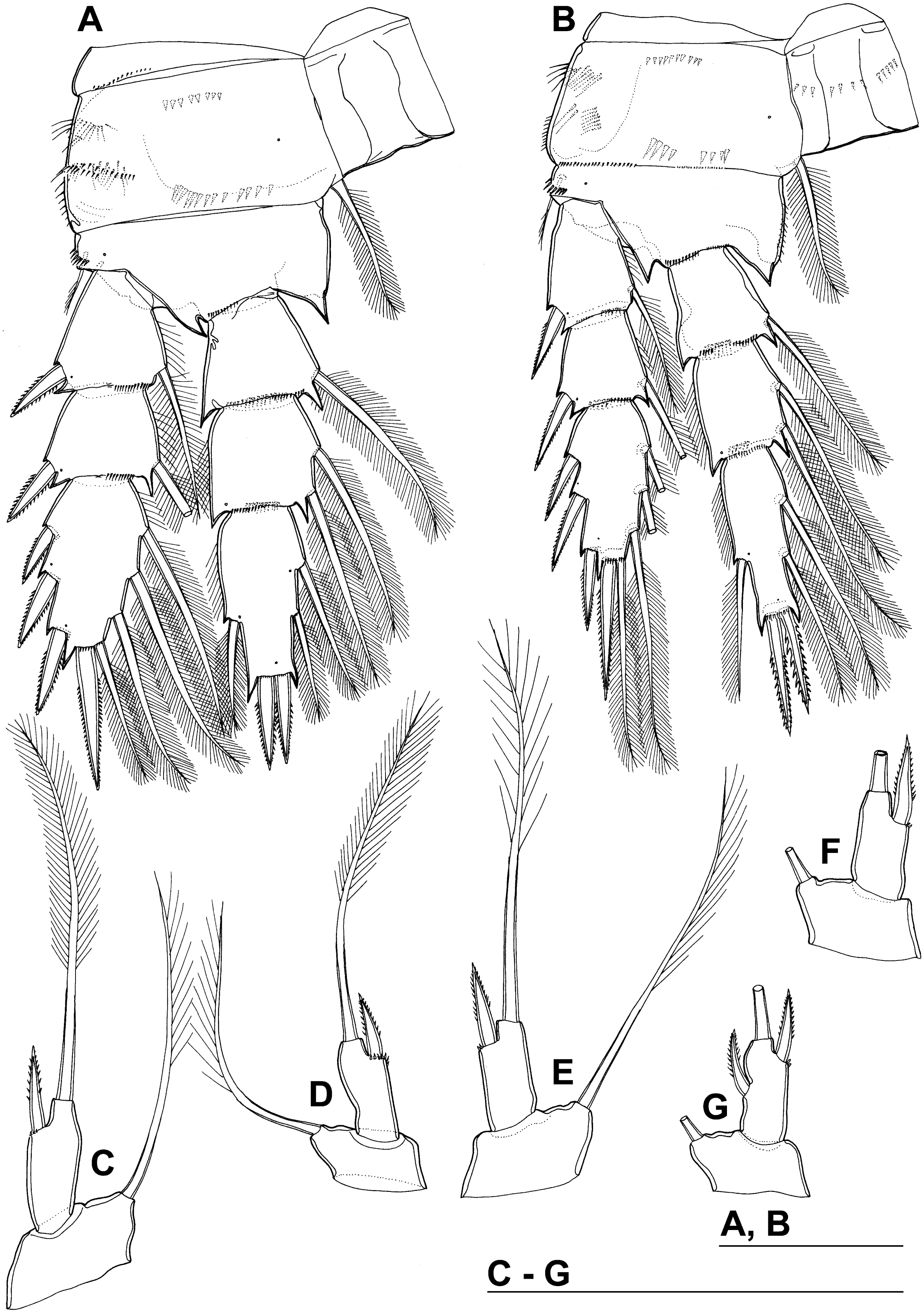

Supplementary Figure S17. Line drawings of the female fourth swimming leg (A & B) and fifth leg (C – G), all in anterior view. (**A & C**) *Acanthocyclops europensis*, Sousedovice; (**B & D – G**) *A. vernalis*, Sousedovice. Scales 100 µm. Note: the fourth leg of *A. europensis* (A) is repeated from Fig. 1, but without landmarks and other markings.

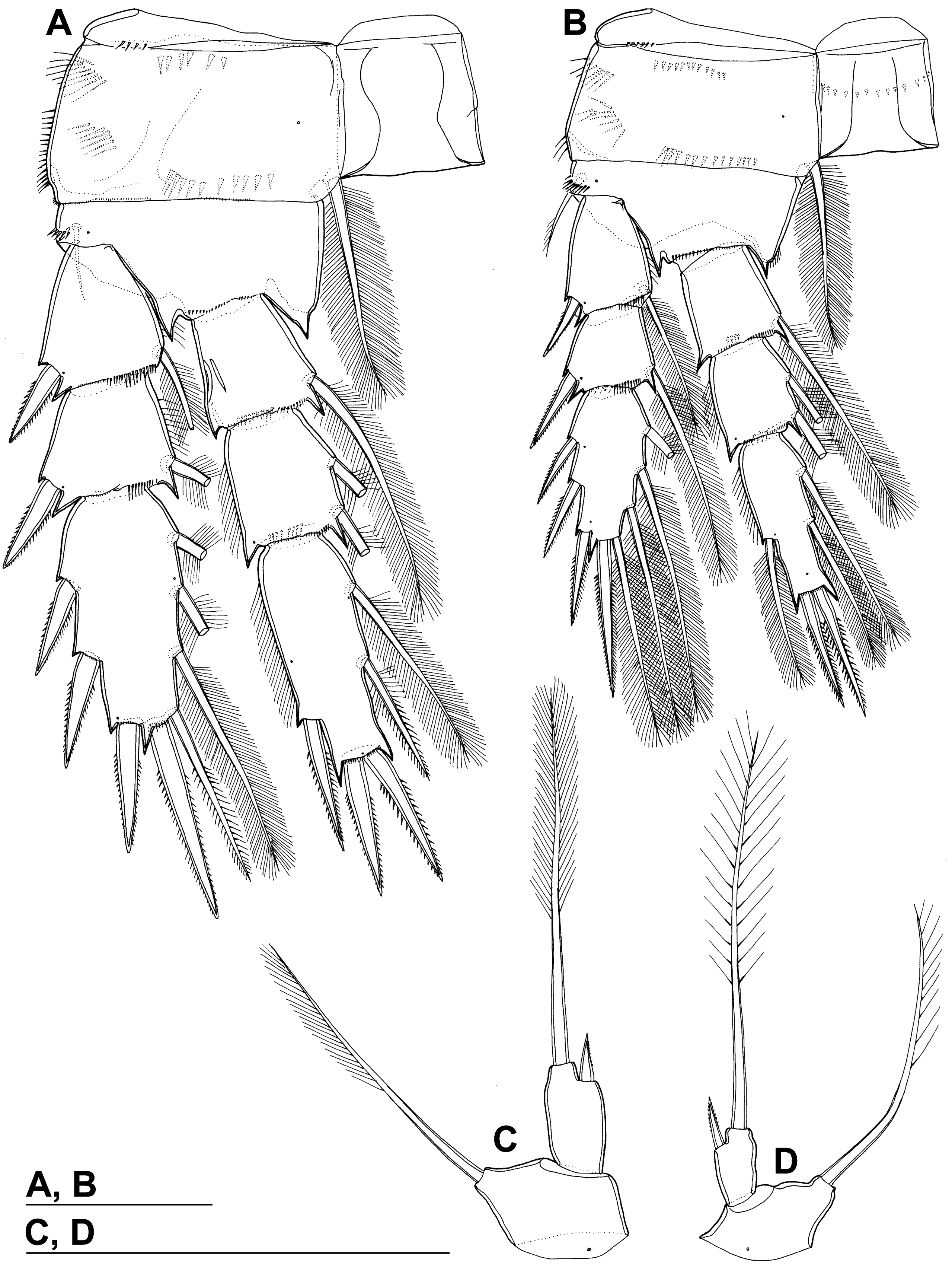

Supplementary Figure S18. Line drawings of the female fourth swimming leg (A & B) and fifth leg (C & D), all in anterior view. (**A & C**) *Acanthocyclops robustus*, Cicenice; (**B & D**) *A. americanus*, Outrata. Scales 100 µm.

SUPPLEMENTARY TABLES:

Supplementary Table S1. Pairwise model-corrected DNA distances within (diagonal) and between four species of *Acanthocyclops* and three outgroup taxa. Concatenated dataset includes CytB, 12S, and ITS-1 sequences. *Macrocylops albidus* (Jurine, 1820) was used as an outgroup to root our concatenated tree (Fig. 2), while *Microcyclops varicans* (Sars, 1863) and *Cyclops insignis* Claus, 1857 were used as outgroups for rooting our COI tree (Supplementary Figure S2).

Taxon	Dataset	*europensis*	*vernalis*	*robustus*	*americanus*	*Macrocyclops*	*Microcyclops*	*Cyclops*
europensis	cyt b	0.01						
	12S	0.01						
	ITS-1	0.01						
	Concatenated	0.01						
	COI	0.02						
vernalis	cyt b	0.22	0.00					
	12S	0.22	0.00					
	ITS-1	0.11	0.00					
	Concatenated	0.20	0.00					
	COI	0.57	0.07					
robustus	cyt b	0.33	0.38	0.00				
	12S	0.26	0.21	0.01				
	ITS-1	0.12	0.09	0.00				
	Concatenated	0.23	0.22	0.00				
	COI	0.66	0.43	0.00				
americanus	cyt b	0.35	0.31	0.35	0.01			
	12S	0.21	0.22	0.20	0.01			
	ITS-1	0.15	0.15	0.11	0.00			
	Concatenated	0.25	0.25	0.20	0.01			
	COI	0.63	0.35	0.42	0.01			
Macrocyclops	cyt b	0.31	0.81	0.81	0.23	-		
	12S	0.60	0.60	0.60	0.60	-		
	ITS-1	0.38	0.44	0.31	0.38	-		
	Concatenated	0.93	0.88	0.84	0.89	-		
Microcyclops	COI	1.00	0.52	0.51	0.60	-	0.01	
Cyclops	COI	0.81	0.77	0.72	0.71	-	0.59	0.00

Supplementary Table S2. Percentage of total variance for the first ten eigenvectors (PCs) from the principal component analysis (PCA), with and without allometry. Abbreviations for morphological structures (datasets) as in Fig. 1 and Table 1. Only the symmetric component is shown for the structures with object symmetry (Gs and GsCo). Note that the higher values for P4Bp and GsCo (compared to P4CxBp and Gs respectively) are partly a consequence of reduced dimensionality.

		PC 1	PC 2	PC 3	PC 4	PC 5	PC 6	PC 7	PC 8	PC 9	PC 10
P4Exp3	Uncorrected	45.860	26.847	8.187	5.610	3.402	2.162	1.759	1.630	1.070	0.956
	Size-corrected	53.312	25.242	6.334	4.526	3.019	1.859	1.503	1.139	0.850	0.620
P4Enp3	Uncorrected	53.315	29.603	8.343	2.304	1.930	1.376	1.174	0.899	0.644	0.413
	Size-corrected	56.692	29.996	7.002	2.163	1.269	0.911	0.845	0.529	0.347	0.246
P4CxBp	Uncorrected	24.212	14.942	12.950	8.368	6.685	4.574	3.609	2.897	2.782	2.507
	Size-corrected	32.283	14.501	12.458	7.577	5.010	3.731	3.200	2.911	2.577	2.160
P4Bp	Uncorrected	40.031	13.970	11.034	8.430	7.398	5.840	3.800	2.914	2.519	1.989
	Size-corrected	42.605	18.892	9.899	7.308	6.811	4.689	3.026	2.294	1.757	1.316
Cr	Uncorrected	46.167	17.744	13.965	8.646	5.136	3.410	1.832	1.558	1.062	0.480
	Size-corrected	39.301	25.677	18.771	5.046	4.533	2.709	1.568	1.204	0.758	0.433
Gs	Uncorrected	34.993	24.712	13.427	5.661	4.752	4.091	2.601	1.826	1.197	1.059
	Size-corrected	36.107	24.508	12.507	5.984	5.026	3.500	2.643	1.884	1.258	1.071
GsCo	Uncorrected	41.166	21.773	11.159	6.756	5.903	4.900	3.981	2.830	1.533	n/a
	Size-corrected	41.217	22.377	11.079	6.210	6.097	4.637	3.991	2.889	1.503	n/a

Supplementary Table S3. Overall strength of association between datasets, estimated by RV coefficients from the partial least squares (PLS) analysis, pooled by species and sex. Abbreviations for morphological structures (datasets) as in Fig. 1 and Table 1. P-value was estimated after 10,000 permutations against the null hypothesis of complete independence, using the RV coefficient as the test statistic. The covariation for the first ten PLS axes is given as a percentage of total covariation, and the last PLS axis with a significant P-value is marked with an asterisk. No. – number of individuals matched in both datasets.

	No.	RV	P	PLS 1	PLS 2	PLS 3	PLS 4	PLS 5	PLS 6	PLS 7	PLS 8	PLS 9	PLS 10
P4Enp3-P4Exp3	119	0.3035	<.0001	77.075	10.644	5.354	3.896	*1.999	0.459	0.266	0.209	0.084	0.014
P4Enp3-P4CxBp	140	0.0981	0.0015	44.990	25.753	11.394	*8.014	3.871	2.488	1.586	1.234	0.546	0.124
P4Enp3-Gs	93	0.0983	0.0073	66.390	*23.299	3.670	3.169	1.492	1.139	0.455	0.264	0.082	0.039
P4Enp3-Cr	140	0.0723	0.0039	60.221	19.536	10.294	5.755	2.510	*1.430	0.092	0.083	0.057	0.023
P4Exp3-P4CxBp	123	0.1150	0.0007	41.069	20.046	13.610	8.550	*6.826	3.238	2.657	1.162	1.007	0.654
P4Exp3-Gs	85	0.0837	0.0718	57.809	20.907	9.509	4.075	3.268	2.489	0.656	0.540	0.386	0.174
P4Exp3-Cr	124	0.0618	0.0450	45.368	33.717	7.785	6.615	3.319	1.918	0.605	0.362	0.220	0.092
P4CxBp-Gs	97	0.1074	0.0104	*61.591	21.345	6.449	2.539	2.255	1.731	1.257	0.612	0.576	0.391
P4CxBp-Cr	144	0.0614	0.0736	51.614	21.744	10.765	6.679	3.436	2.399	1.492	1.175	0.512	0.182
Gs-Cr	100	0.1201	0.0004	67.475	23.649	*6.457	0.875	0.580	0.467	0.208	0.158	0.095	0.036

Supplementary Table S4. Individual variation vs fluctuating asymmetry matrix correlation, both overall and pooled by species and sex. Abbreviations for morphological structures (datasets) as in Fig. 1 and Table 1. P-value is calculated after 10,000 permutation rounds.

	Pooled by species and sex				Overall			
	With diagonals		Without diagonals		With diagonals		Without diagonals	
	Index	P	Index	P	Index	P	Index	P
P4Exp3	0.8982	<.0001	0.8004	<.0001	0.6784	<.0001	0.5713	<.0001
P4Enp3	0.9108	0.0001	0.7777	0.0002	0.8263	0.0020	0.6093	0.0012
P4CxBp	0.9540	<.0001	0.9107	<.0001	0.7766	<.0001	0.5472	<.0001
P4Bp	0.9439	<.0001	0.8452	<.0001	0.7456	0.0005	0.4145	0.0027
Cr	0.6018	0.0212	0.4124	0.0385	0.5262	0.1394	0.3207	0.1213
Gs symmetric	0.2249	<.0001	0.1293	<.0001	0.2493	0.0001	0.1394	0.0004
Gs asymmetric	0.9918	<.0001	0.9912	<.0001	0.9887	<.0001	0.9876	<.0001
GsCo symmetric	0.8426	0.0389	0.0992	0.0649	0.6224	0.2054	0.1443	0.1934
GsCo asymmetric	0.9993	<.0001	0.9988	<.0001	0.9987	<.0001	0.9975	<.0001

Supplementary Table S5. Percentages of correctly identified specimens in species pairs of *Acanthocyclops* through discriminant function analysis (DFA), after cross-validation. Abbreviations for morphological structures (datasets) as in Fig. 1 and Table 1. P-value, both parametric and after 10,000 permutation runs, was highly significant in all analyses (<.0001), but the results for Gs should be interpreted with caution because of a small sample size relative to the dimensionality of data.

Dataset	*americanus - europensis*	*americanus - robustus*	*americanus - vernalis*	*europensis - robustus*	*europensis - vernalis*	*robustus - vernalis*
P4Exp3	100% - 100%	100% - 100%	100% - 100%	100% - 100%	92% - 100%	100% - 100%
P4Enp3	100% - 100%	100% - 100%	100% - 100%	100% - 100%	93% - 100%	100% - 100%
P4CxBp	100% - 100%	100% - 100%	100% - 100%	100% - 100%	85% - 90%	100% - 100%
Cr	96% - 100%	93% - 94%	100% - 100%	92% - 87%	98% - 100%	97% - 98%
Gs	100% - 97%	94% - 95%	65% - 74%	100% - 95%	97% - 100%	75% - 85%
GsCo	100% - 94%	94% - 80%	100% - 96%	100% - 100%	100% - 96%	65% - 85%
P4Bp	100% - 100%	100% - 100%	100% - 100%	100% - 100%	85% - 82%	100% - 100%

Supplementary Table S6. Material sequenced for one nuclear (ITS-1) and three mitochondrial markers (CytB, 12S, COI). The COI partial sequences were analysed in combination with all other COI sequences available from GenBank (Supplementary Fig. S2), while other markers were combined into a concatenated dataset and analysed separately (Fig. 2). Note that some haplotypes were found in more than one place. Populations marked with an asterisk were also used for geometric morphometric analyses (see Supplementary Table S7)

Species	Country	Locality	Habitat	Latitude	Longitude	Date	GenBank accession numbers			
							CytB	12S	ITS-1	COI
europensis	Czech Republic	*Sousedovice	forest pool	49°13'33.0"	13°52'16.5"	21 Mar 2016	MG230137-39	MG230117-18	MG230128-29	MG230151
	Austria	*Lesach	temporary forest pool	46°59'1.8"	12°38'0.4"	9 Jun 2016	MG230140	MG230114-16	MG230127	MG230152
vernalis	Czech Republic	*Vodňany, V korýtkách	temporary pool	49°09'25.2"	14°10'13.2"	26 Apr 2016	MG230142-44	MG230119	MG230130	-
		*Sousedovice	artificial pool	49°13'37.9"	13°52'04.8"	25 Apr 2016	MG230141, 45	MG230119	MG230130	-
robustus	Czech Republic	Lužnice river, Majdalena	river littoral	48°58'21"	14°51' 54"	Nov 2008	MG230136	MG230122	MG230132	MG230153
		Blanice river, Vodňany	river littoral	49°09'23.1"	14°10'10.4"	Nov 2008	-	MG230122	-	-
		Sousedovice	pond littoral	49°13'47.0"	13°52'25.7"	Apr 2016	-	MG230122	MG230132	MG230153
	France	Lindre-Basse	pond littoral	48°47'52.2"	6°44'51.2"	Jun 2009	-	MG230121	MG230132	-
	Denmark	Sjørup	eutrophic pond littoral	56°25'51.1"	9°06'09.6"	12 Aug 2009	MG230135	MG230120	MG230131	-
americanus	Czech Republic	Sousedovice	pond littoral	49°13'47.0"	13°52'25.7"	Apr 2016	-	MG230124	-	MG230155
	France	Lindre-Basse	pond littoral	48°47'52.2"	6°44'51.2"	Jun 2009	MG230146, 50	MG230123, 25	MG230133	MG230154
	Austria	Neusiedler See, Illmitz	shallow lake	47°46'21.3"	16°44'25.3"	Apr 2007	MG230147-49	MG230126	MG230133-34	-
	Spain	Rio Guadiana, Badajoz	river littoral	38°52'26.4"	6°59'37.4"	Oct 2008	-	MG230124	-	MG230156

Supplementary Table S7. Number of specimens examined for LBGM analyses for each structure in the four *Acanthocyclops* species studied. Abbreviations for morphological structures (datasets) as in Fig. 1 and Table 1. Gs is the only dataset with object symmetry; this structure is not present in males. All localities are in the Czech Republic, except for Lesach (Austria). Populations marked with an asterisk were also used for molecular analyses (see Supplementary Table S6), but not the same specimens.

Species	Locality	Latitude	Longitude	Date	P4Exp3		P4Enp3		P4CxBp		Cr		Gs
					♀	♂	♀	♂	♀	♂	♀	♂	♀
europensis	*Sousedovice, forest pool	49°13'33.0	13°52'16.5"	21 Mar 2016	27	12	26	12	27	12	27	12	27
	*Lesach, temporary forest pool	46°59'1.8”	12°38' 0.4”	9 Jun 2016	7	-	9	-	9	-	9	-	9
vernalis	*Sousedovice, artificial pool	49°13'37.9"	13°52'04.8"	25 Apr 2016	19	4	20	4	19	4	20	4	20
	*Vodňany, temporary pool	49°09'25.2"	14°10'13.2"	26 Apr 2016	7	9	7	9	7	9	7	9	7
robustus	Cicenice, small sand pits	49°08'12.3"	14°15'22.2"	29 Apr 2016	10	10	10	11	10	11	10	11	10
	Sousedovice, artificial pool	49°13'37.9"	13°52'04.8"	25 Apr 2016	10	-	10	-	10	-	10	-	10
americanus	Outrata, carp fishpond	49°09'25.0"	14°10'22.7"	20 May 2009	11	10	17	11	17	11	17	11	17
Subtotal					91	45	99	47	99	47	100	47	100
Total					136		146		146		147		100
